# Establishing a functional osteochondral–synovial ex vivo joint model: Systematic evaluation of glucose and oxygen impact

**DOI:** 10.1016/j.jot.2026.101240

**Published:** 2026-10-24

**Authors:** Fatemeh Safari, Jovana Zvicer, Sibylle Grad, Martin J. Stoddart, Zhen Li

**Affiliations:** a AO Research Institute Davos, Davos Platz, Switzerland; b University of Belgrade, Faculty of Technology and Metallurgy, Belgrade, Serbia

**Keywords:** Co-culture, Glucose, Metabolomics, Osteochondral explant, Physioxia, Synovium

## Abstract

**Background:**

Osteochondral–synovial co-culture *ex vivo* models represent an advanced platform for investigating joint biology and disease mechanisms, as they capture key tissue interactions, including bone–cartilage crosstalk and synovial inflammatory regulation. However, the broader application of these models remains limited by the lack of consensus regarding optimal glucose and oxygen levels in *ex vivo* joint models. This study aims to examine how glucose concentration in medium and oxygen availability during the culture impact the maintenance and metabolic activity of tissues in a complex osteochondral–synovial *ex vivo* co-culture model, with the aim of identifying optimal culture conditions suitable for controlled investigation of inter-tissue interactions, inflammatory and degenerative responses, and preclinical assessment of potential therapies prior to validation in human tissues.

**Methods:**

Bovine osteochondral explants and synovial tissue were harvested from the stifle joint and maintained in static co-culture for 7 days. Cultures were established in either high (HG, 4.5 g/L, supra-physiological) or low (LG, 1 g/L, physiological) glucose Dulbecco's Modified Eagle Medium, under hyperoxic (21% O_2_) or physioxic (5% O_2_) conditions. Tissue responses were assessed independently for each tissue, using cell viability assays, glycosaminoglycan and nitric oxide release measurements, histological analysis, gene expression profiling, and untargeted metabolomics.

**Results:**

LG culture conditions increased cell death in the deep zone of cartilage and in subchondral bone compared with HG medium. Gene expression and metabolomics analyses revealed tissue-specific responses to glucose and oxygen. In cartilage and bone, glucose-driven transcriptional and metabolic changes occurred under hyperoxia but were largely absent under physioxia. In synovium, gene-specific sensitivity to glucose and oxygen was also observed; however, glucose-dependent metabolic responses persisted under physioxia.

**Conclusion:**

This systematic evaluation of glucose and oxygen combinations across cartilage, subchondral bone, and synovium demonstrates that, HG medium under physioxic conditions best preserves tissue viability and stabilizes molecular responses, particularly in osteochondral tissues. These findings provide evidence-based guidance for optimizing static *ex vivo* joint models and improving their reproducibility and translational value.

**The translational potential of this article:**

This study established optimized culture conditions for a bovine osteochondral–synovial *ex vivo* co-culture model, improving its translational relevance for the controlled investigation of tissue responses and preclinical screening of drugs and biomaterials prior to validation in human models.

## Graphical abstract

**Figure:**
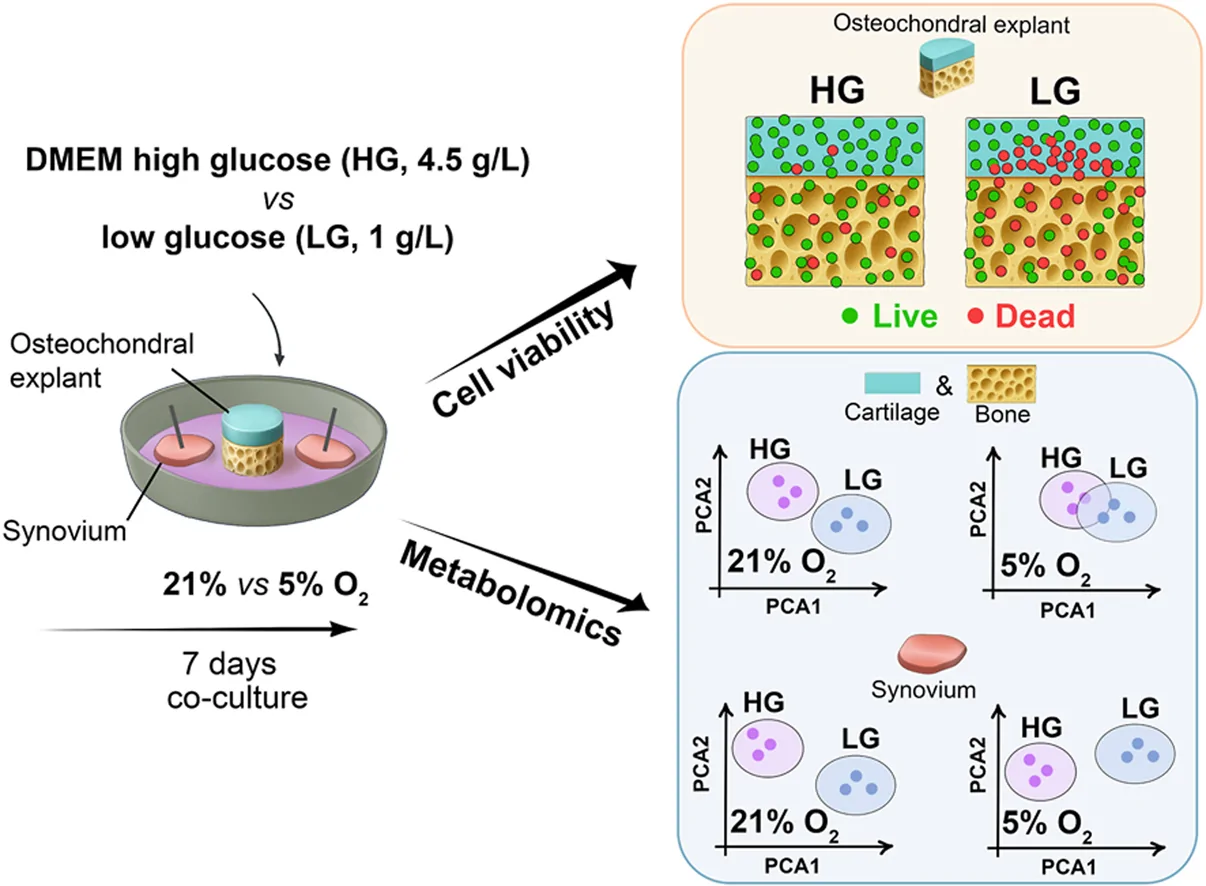

## 1 Introduction

*Ex vivo* explant-based models occupy a unique position between simplified *in vitro* systems and complex *in vivo* animal studies [1,2]. By preserving native extracellular matrix (ECM) architecture and maintaining authentic cell–cell and cell–matrix interactions, they capture biological features that are lost in monolayer cultures or even in advanced 3D constructs such as pellets or organoid models [3,4].

For articular cartilage regeneration, *ex vivo* models have been widely applied in the functional assessment of emerging therapies [[5], [6], [7]]. Beyond regenerative applications, these systems play a central role in osteoarthritis (OA) research, allowing investigations of cartilage degeneration, enabling controlled disease-like environments, and helping in pre-screening of therapeutic candidates [8]. Since OA affects hundreds of millions of patients worldwide and remains without disease-modifying treatments, the possibility to assess therapeutic responses in a mechanistically relevant system is of major clinical importance.

To create an *ex vivo* environment that more accurately reflects *in vivo* joint biology and enhances the translational value of preclinical findings, one strategy is to combine osteochondral explants with synovial tissue [1]. Osteochondral explants already outperform cartilage only models, as the presence of subchondral bone markedly alters inflammatory and catabolic signaling and improves chondrocyte survival, likely through the release of bone-derived mediators [9]. Including synovial tissue further enhances the physiological relevance of the model, as recent studies have demonstrated that synovial tissue macrophages (STMs) form an immune-protective lining barrier that regulates joint inflammation and may control disease progression or remission in inflammatory joint disorders, such as rheumatoid arthritis [10]. It is known that synovial inflammation strongly correlates with OA pain and progression. Therefore, incorporating synovium enables modeling of clinically relevant inflammatory pathways that are not captured in cartilage-only explants.

Although this integrated osteochondral–synovium model provides a robust foundation for complex *ex vivo* joint models, the field still lacks standardized protocols. This gap is particularly evident in the definition of two fundamental culture parameters, glucose availability and oxygen concentration, which are critical for maintaining tissue physiology. It is generally recognized that all three joint tissues operate under markedly reduced oxygen conditions, with cartilage experiencing gradients from approximately 6% in the superficial zone to as low as 1% in the deep zone [11]. Vascularized tissues such as bone and synovium exhibit higher levels of oxygen between 5% and 12.5% [12,13]. Physiological glucose concentrations in cartilage, bone and synovial fluid are substantially lower than those present in standard high glucose (HG, 4.5 g/L, ∼25 mM) media and are typically around 5 mM (∼0.9 g/L) [14,15], which is in the range of normal blood glucose.

However, studies have shown contrasting results regarding tissue metabolism and its maintenance *in vitro*, depending on whether physiological conditions (low glucose, LG, ∼5 mM glucose and 5% O_2_ physioxia) or non-physiological conditions (∼25 mM glucose and 21% O_2_ hyperoxia) were used. It is shown that physioxia promotes cartilage matrix synthesis by enabling direct binding of HIF-2α to a regulatory site in SOX9 gene, while suppressing cartilage degradation through predominantly HIF-1α–mediated anti-catabolic responses [16]. In bone tissue, low oxygen levels exert a dual HIF-dependent effect, where HIF-1α promotes anabolic responses while on contrary HIF-2α limits further catabolic effects by inhibiting osteoblast differentiation and bone mineralization [17]. These findings are further supported by an *ex vivo* study using porcine osteochondral explants, which demonstrated enhanced cartilaginous matrix deposition in the defect site under 2% oxygen [18]. Furthermore, an *in vivo* study in rats maintained under 12% oxygen conditions demonstrated enhanced cartilage matrix formation and improved structural organization within cartilage defects after 4 weeks, compared with animals maintained under 21% oxygen [19].

Regarding glucose availability, concentrations above the physiological level (5 mM) had adverse effects on synovium, cartilage, and bone. In synovium explants, higher glucose levels (12.5 mM) reduced DNA content, collagen levels, and glycosaminoglycans (GAG) deposition compared to physiological concentrations (5 mM) [20]. In addition, in bone, acute exposure to HG (25 mM) enhanced glycolysis and oxidative phosphorylation, and chronic exposure resulted in uncoupled oxygen consumption, increased ROS generation, downregulation of key mitochondrial bioenergetic proteins, and consequently elevated oxidative stress [21]. Despite these adverse effects, different studies have demonstrated that standard laboratory culture conditions, typically employing HG DMEM and atmospheric oxygen levels (21% O_2_), can maintain cartilage viability and structural integrity even during long-term culture [22]. These inconsistencies hinder the reproducibility of preclinical studies and limit the translation of *ex vivo* findings to clinical contexts. Although human tissues are essential for clinical translation, their availability, frequent presence of osteoarthritic degeneration, and limited access to matched synovial tissue from the same patient restrict their use in controlled methodological studies. In contrast, bovine explants are well established in cartilage research and have been used to investigate extracellular-matrix turnover, chondrocyte function, formation of the calcified cartilage zone, and controlled cartilage damage for the assessment of emerging repair therapies [[23], [24], [25]]. Thus, they provide a reproducible platform for optimizing culture conditions and screening potential treatments before validation in human tissues or *in vivo* models.

Given the lack of standardized protocols and data driven guidance on optimal culture conditions for *ex vivo* models of the articular joint, this study evaluated the combined effects of glucose concentration (LG DMEM *vs.* HG DMEM) and oxygen availability (hyperoxic *vs.* physioxic) in a complex bovine osteochondral–synovial co-culture model (Fig. 1). By comparing cell viability, gene expression, and metabolomic profiles across cartilage, bone, and synovium, we aimed to identify culture conditions that maintain joint tissue viability and cell phenotype *ex vivo*, thereby providing evidence-based recommendations for future joint research using *ex vivo* models.

**Fig. 1:**
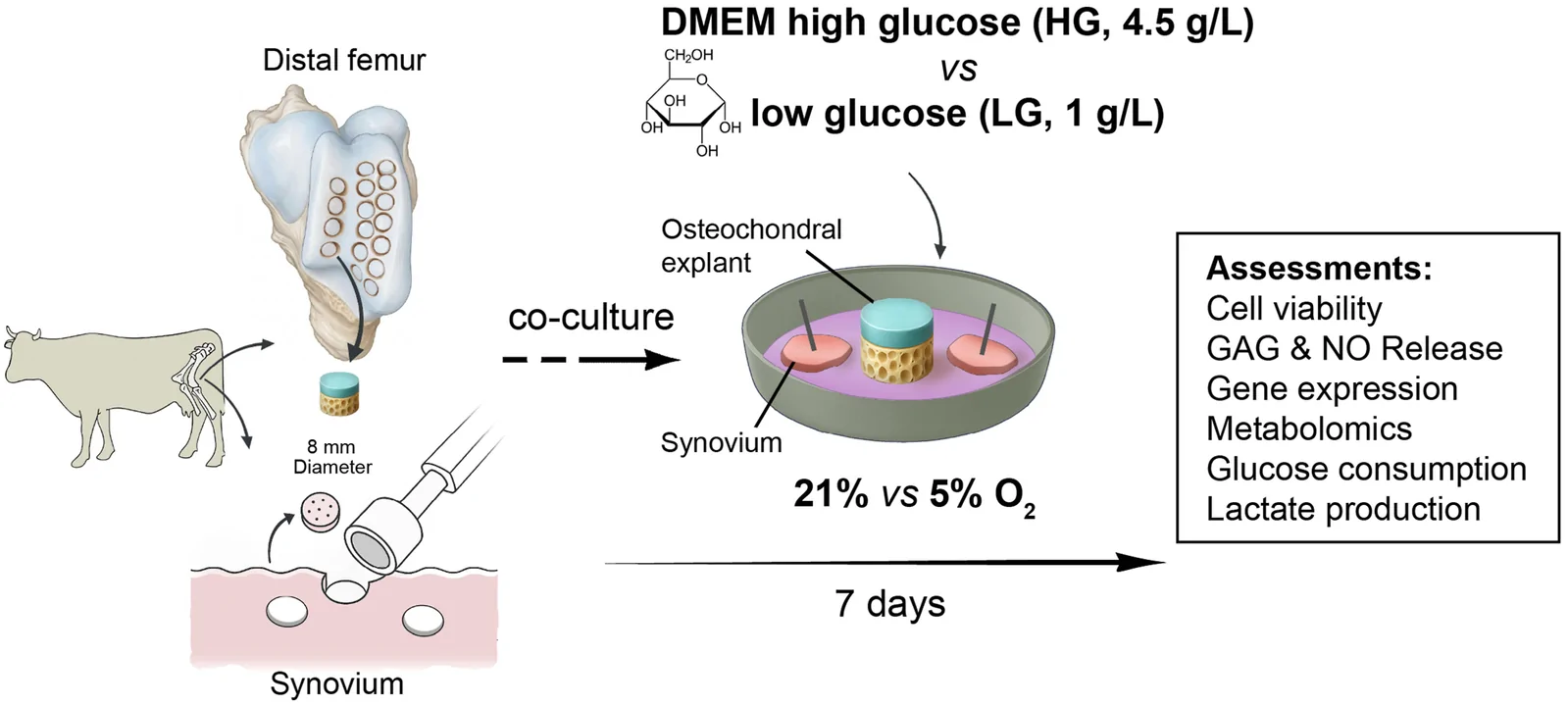
Schematic representation of experimental design.

## 2 Materials and methods

### 2.1 Tissue isolation and culture

Bovine stifle joints were obtained from a local abattoir (Metzgerei Angst AG, Zurich, CH) within 48 h of slaughter (3 males and 2 females). Cylindrical osteochondral explants (8 mm in diameter, 6 mm in height) were collected from the femoral groove as previously described [26] (Fig. 1). Synovium tissues were collected from the same joint and 8 mm diameter discs were obtained using a biopsy punch (KAI medical, Seki-shi, Gifu Prefecture). Both osteochondral and synovium tissue biopsies were transferred to phosphate-buffered saline (PBS) supplemented with 10% penicillin/streptomycin (P/S, Gibco) for at least 20 min. Every tissue sample was then rinsed once with PBS supplemented with 1% P/S and transferred to a 24-well plate containing 2 ml of Dulbecco's modified eagle medium (DMEM-HG, 4.5 g/L-glucose; Gibco) supplemented with 10% fetal bovine serum (FBS, Gibco) and 1% P/S. At this time, for metabolomics analysis, tissues were preserved and designated as control fresh tissue. After overnight incubation at 37°C, 21% O_2_ and 5% CO_2_, co-cultures of osteochondral plugs with synovium tissues were started using either DMEM HG or DMEM LG (1 g/L glucose; Gibco) supplemented with 1% P/S, 1% insulin-transferrin-selenium (ITS) + Premix (Corning, Tewksbury, MA, USA), 1% non-essential amino acids (NEAA, Gibco), 50 μg/mL ascorbic acid (Sigma–Aldrich), and 2.5% HEPES (Gibco). To stabilize the osteochondral tissue at the center of the custom-made tissue holders (made of Polyether ether ketone (PEEK), ∼31 mm diameter and ∼10 mm height) and to prevent cell migration from the subchondral bone, 800 μL of 2% w/v agarose gel was applied. After gel solidification, two synovial tissue punches were placed into the tissue holder. Synovium tissues were secured using a 26G needle (Sterican, 0.45 × 25 mm) anchored to the bottom of the holder. The excess portion of the needle was removed using sterile tin snips. At this point, some tissues were preserved and designated as control day 0 for gene expression analysis. *Ex vivo* co-cultures were conducted either in the normal incubator containing 21% O_2_ or in a Hypoxia/Physioxia Workstation (Baker Ruskinn) containing 5% O_2_ for 7 days. To maintain stable oxygen level in culture media throughout the experiment, culture media for the 5% O_2_ groups were pre-equilibrated inside the Hypoxia/Physioxia workstation for at least 10 h prior to each media change. During equilibration, the medium was kept in an open sterile bottle to allow gas exchange with the workstation atmosphere.

### 2.2 Cell viability assays

Cell viability was evaluated using live/dead staining by Lactate Dehydrogenase (LDH)/Ethidium homodimer-1 (EthHD1) staining for the osteochondral tissues and calcein AM/EthHD1 staining for synovium. For this, on day 7 of culture, osteochondral plugs were cut in 2 halves. One half was transferred to cryo-compound and then snap frozen in liquid nitrogen. After cross-sectioning using cryo-tape (Cryofilm type 3C, SECTION-LAB Co) for non-decalcified tissues, 8 μm cuts of osteochondral tissues were stained using LDH/EthHD1 according to the protocol [27]. Cell viability was then evaluated using Olympus microscopy. For synovium tissue, fresh samples were stained using calcein AM/EthHD. In brief, freshly prepared solution of 4 μM calcein AM (Sigma–Aldrich) and 2.3 μM EthHD1 (Sigma–Aldrich) in PBS was used. Synovium tissues were incubated in this solution for 1 h at 37°C. Cell viability was evaluated using confocal microscopy (LSM 800; Carl Zeiss AG).

QuPath (0.7.0 version) was used to analyze the microscopy images. For cartilage sections the central part and for subchondral bone sections the central and edge parts of every cross-section were considered for cell counting process as indicated in Fig. 2. For synovium tissue, five similar regions of each image were analyzed. The number of dead cells and live cells was counted manually in all images. Four to five biological replicates were used for semi-quantification and statistical analysis.

**Fig. 2:**
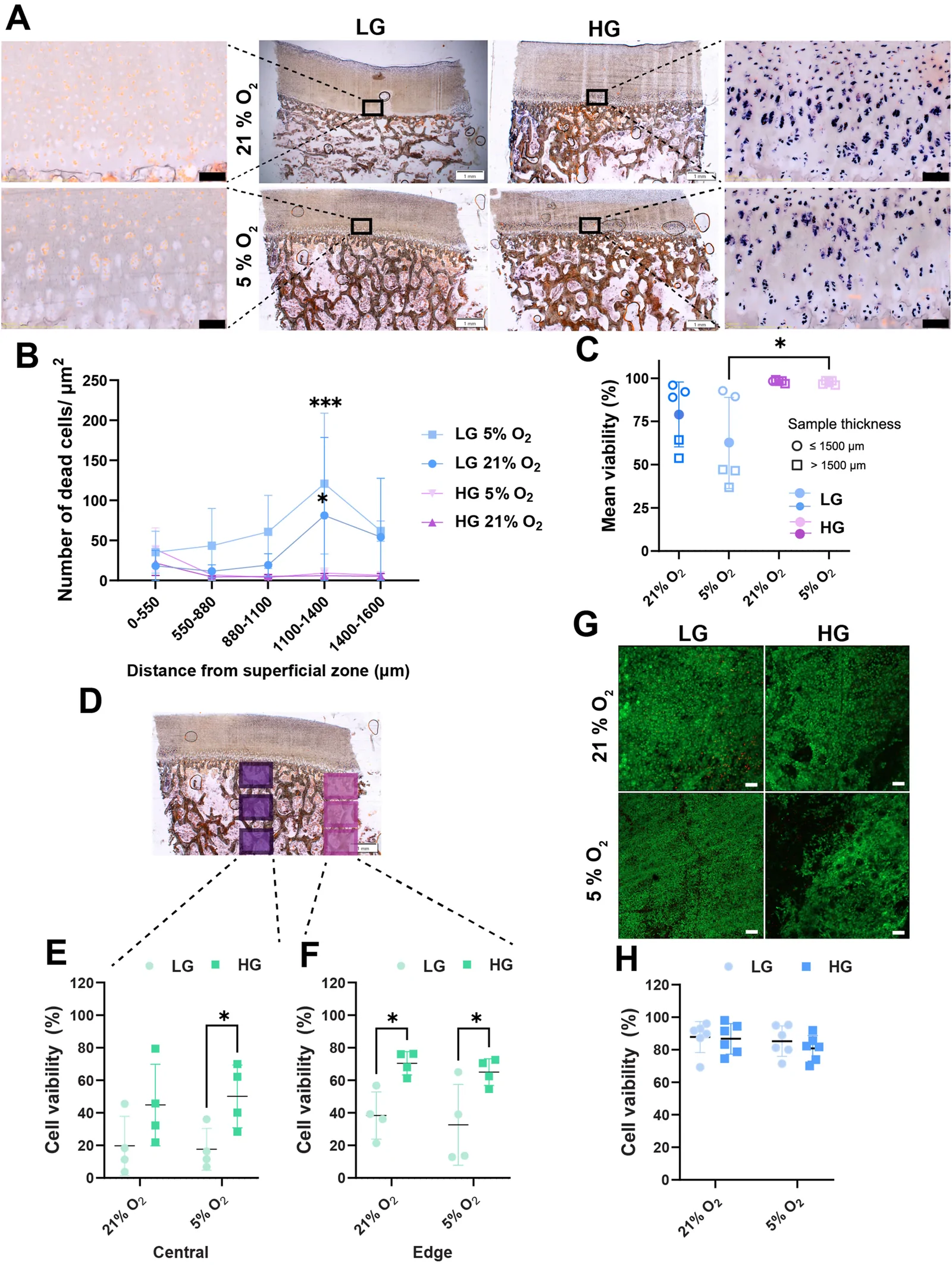
Cell viability of joint tissues. Co-cultures of osteochondral and synovium tissues were performed for 7 days in low glucose (LG) and high glucose (HG) DMEM under physioxia (5% O_2_) and hyperoxia (21% O_2_). (A) LDH/Ethidium homodimer staining in osteochondral tissues (scale bars in full osteochondral samples = 1 mm, in the higher magnification = 100 μm). (B) Average number of dead cells per counted area in different regions of cartilage (significancy was calculated between LG and HG either under 5% or 21% O_2_), and (C) average cell viability in full thickness of cartilage (significancy is calculated using Kruskal–Wallis test followed by Dunn's multiple comparisons test). (D-F) Cell viability in subchondral bone was evaluated according to the pattern in figure (D) after LDH/Ethidium homodimer staining in (E) the central region and (F) the peripheral region of the subchondral bone. (G) Calcein/ethidium homodimer staining was performed for evaluation of cell viability in (H) synovium tissue (scale bar = 100 μm). Results are presented as means of 5 donors for cartilage, 4 donors for bone, and 6 donors for synovium. Statistical analysis was performed using 2-way ANOVA, with significance levels indicated as *p < 0.05, **p < 0.01, ***p < 0.001, ****p < 0.0001.

### 2.3 Analysis of gene expression by real-time quantitative reverse transcription PCR (RT-qPCR)

Osteochondral tissues from the control group (day 0) and from the experiments (day 7) were first separated into cartilage and bone. Half of the osteochondral explant was used for RNA isolation. All three tissues, including synovium were cut into small pieces using scalpel and were immediately snap-frozen. For RNA isolation, each sample was pulverized using a house-made metal tool and liquid nitrogen. To keep the sample frozen during the pulverization, all the tools were pre-cooled before. Samples were then transferred to a 2-mL Eppendorf tube containing 1 mL TRI reagent (Molecular Research Center) and further homogenized using a 6 mm diameter metal ball and tissue homogenizer for 3 min at 30 Hz frequency for 2-3 times for tissue homogenization. RNA isolation and cDNA synthesis were performed using the RNeasy Mini Kit (Qiagen) and Superscript Vilo RT kit (Thermo Fisher Scientific), respectively. RT-qPCR was then performed using TaqMan Gene Expression Master Mix with QuantStudio Flex 7 instrument. Used primers and probes are listed in Table 1. Gene expression assays from Applied Biosystems were used for additional genes including: *IL8* (Bt03211906_m1), *ALPL* (Bt03244508_m1), *RPLP0* (Bt03218086_m1), *GLUT1* (Bt03215313_m1), *GLUT2* (Bt03258678_m1), *GLUT3* (Bt03259519_gH), *GLUT4* (Bt03215316_m1), *GLUT5* (Bt03258292_m1), *BGLAP* (Bt03213535_g1), and *VEGFA* (Bt03213282_m1).

**Table 1:** Custom designed primers and probes used for qRT-PCR.

Gene name	Forward primer seq	Reverse primer seq	Probe seq
*COL1A2*	5'-TGC AGT AAC TTC GTG CCT AGC A-3'	5'-CGC GTG GTC CTC TAT CTC CA-3'	5'-CAT GCC AAT CCT TAC AAG AGG CAA CTG C-3'
*COL2A1*	5′-AAG AAA CAC ATC TGG TTT GGA GAA A-3′	5′-TGG GAG CCA GGT TGT CAT C-3′	5′-CAA CGG TGG CTT CCA CTT CAG CTA TGG-3′
*COL10A1*	5′-ACT TCT CTT ACC ACA TAC ACG TGA AAG-3′	5′-CCA GGT AGC CCT TGA TGT ACT CA-3′	5′-TGC CGT TCT TAT ACA GAC CTA CCC AAG CAT G-3′
*ACAN*	5′-CCA ACG AAA CCT ATG ACG TGT ACT-3′	5′-GCA CTC GTT GGC TGC CTC-3′	5′-ATG TTG CAT AGA AGA CCT CGC CCT CCA T-3′
*IL6*	5′-TTC CAA AAA TGG AGG AAA AGG A-3′	5′-TCC AGA AGA CCA GCA GTG GTT-3′	5′CTT CCA ATC TGG GTT CAA TCA GGC GAT T-3′
*MMP1*	5′-TTC AGC TTT CTC AGG ACG ACA TT-3′	5′-CGA CTG GCT GAG TGG GAT TT-3′	5′-TCC AGG CCA TCT ACG GAC CTT CCC-3′
*MMP3*	5′-GGC TGC AAG GGA CAA GGA A-3′	5′-CAA ACT GTT TCG TAT CCT TTG CAA-3′	5′-CAC CAT GGA GCT TGT TCA GCA ATA TCT AGA AAA C-3′
*MMP13*	5′-CCA TCT ACA CCT ACA CTG GCA AAA G-3′	5′-GTC TGG CGT TTT GGG ATG TT-3′	5′-TCT CTC TAT GGT CCA GGA GAT GAA GAC CCC-3′
*ADAMTS4*	5′-CCC CAT GTG CAA CGT CAA G-3′	5′-AGT CTC CAC AAA TCT GCT CAG TGA-3′	5′-AGC CCC CGA AGG GCT AAG CGC-3′
*ADAMTS5*	5′-GAT GGT CAC GGT AAC TGT TTG CT-3′	5′-GCC GGG ACA CAC CGA GTA C-3′	5′-AGG CCA GAC CTA CGA TGC CAG CC-3′
*COMP*	5′-CCA GAA GAA CGA CGA CCA GAA-3′	5′-TCT GAT CTG AGT TGG GCA CCT T-3′	5′-ACG GCG ACC GGA TCC GCA A-3′

### 2.4 GAG and NO measurement

For evaluation of the glycosaminoglycan (GAG) and nitric oxide (NO) release, conditioned medium was collected every day for 7 days of culture and kept at −20°C till analysis. The amount of GAG released into the medium was determined by the dimethyl-methylene blue (DMMB, Sigma–Aldrich) dye method and analyzed using published methodology protocol [28]. Nitric oxide (NO) released in medium was quantified using the Griess Reagent System (Promega Corporation) according to the manufacturer's protocol. Absorbance for both analyses were recorded at 535 nm using the TECAN microplate reader.

### 2.5 Histology

For Safranin O/Fast Green staining, cryo-sections, which were prepared as described in the Cell viability assays section, were fixed in 4% formalin (Formafix AG), washed in distilled water (dH2O) to remove the cryo compound, then stained with Weigert's Haematoxylin solution (Sigma–Aldrich) for 30 min and rinsed in tap water for 10 min. The slides were then stained with 0.02% Fast Green (Fluka #51275) for 6 min and washed in 1% acetic acid for 30 s. Then they were stained with 0.1% Safranin-O (Sigma–Aldrich) for 12 min. Subsequently, samples were rinsed with dH_2_O. After dehydration with increasing concentrations of ethanol, samples were transferred to xylene and cover-slipped with Eukitt mounting medium (Sigma–Aldrich). Safranin O/Fast Green staining was performed as a qualitative assessment of tissue morphology and proteoglycan distribution. No histological scoring system was applied.

### 2.6 Sample preparation for LC-MS analysis

Untargeted metabolomics was performed by the Functional Genomics Center Zurich (ETH Zurich/University of Zurich, Switzerland) on *Bos taurus* bones, cartilage and synovium samples, separately. Frozen tissues were cryopulverized and the polar metabolites extracted with ice-cold methanol:water (80:20, v/v). Fresh tissues were used as control. Homogenates were centrifuged for 10 min at 14000 rpm/4°C and metabolite extracts in supernatant were transferred to clean test tubes. Prior to measuring, extracts were dried under nitrogen flow and reconstituted in injection buffer (90% acetonitrile). The solution was vortexed and centrifuged for 10min at 14000 rpm/4°C and 60 mL of the supernatants were transferred to glass vials with narrowed bottom (Total Recovery Vials, Waters) for liquid chromatography and mass spectrometry (LC-MS) injection. In addition, method blanks, quality control (QC) standards, and pooled samples were prepared in the same way to serve as quality control for the measurements.

### 2.7 LC-MS analysis

Polar metabolites were separated on a Thermo Vanquish Horizon Binary Pump equipped with Waters Premier BEH Amide column (150 mm × 2.1 mm), applying a gradient of 10 mM ammonium bicarbonate in 5% acetonitrile (A) and 10 mM ammonium bicarbonate in 95% acetonitrile (B) from 99% B to 30% B over 12 min. The injection volume was 4 mL. The flow rate was 0.4 μL/min with a column temperature of 40°C and autosampler temperature of 5°C.

The LC was coupled to Thermo Exploris 480 mass spectrometer by a HESI source. MS1 (molecular ion) and MS2 (fragment) data were acquired using negative polarization and Full MS/dd-MS^2^ (Top5) over a mass range of 70 to 1050 m/z at MS1 resolution of 60000 and MS2 resolution of 7500.

### 2.8 Untargeted metabolomics data analysis

The metabolomics data set was evaluated in an untargeted fashion with Compound Discoverer software (Thermo Scientific). The modular data analysis workflow includes spectra selection, retention times alignment, compound detection and grouping, gap filling, background filtering, and normalization (data are constant median normalized). mzCloud and mzVault were used to score fragmentation patterns and assign MS2-based identities to the features. A filtering process was performed, leading to the manually annotated compound table, where each feature is annotated with the highest level of confidence. Filtering parameters used were the following: Signa/noise > 3, mzCloud or mzVault match > 50, ppm mass error within ± 5 ppm., match with in-house developed MS1_RT library within ± 10 s, chromatographic peak and MS2 spectra quality. Quality controls were run on pooled QC samples and reference compound mixtures to determine technical accuracy and stability.

Prior to downstream data analysis, metabolite intensity values for each tissue sample were normalized to the total protein content (determined on the protein pellet resulting from polar metabolites extraction). The resulting annotated metabolite table was subsequently imported into MetaboAnalyst version 6.0 for downstream analysis. To improve data comparability and reduce heteroscedasticity, metabolite intensities were log-transformed, followed by autoscaling (mean-centering). This preprocessing approach ensured that metabolites with different dynamic ranges contributed equally to multivariate analyses. The principal component analysis (PCA) was performed separately for each tissue type to visualize global metabolic differences and clustering patterns between experimental groups. PCA was further used as an exploratory tool to assess overall variability and identify potential group-level separation in metabolomic profiles. Student's t-test was used to evaluate differences in metabolite levels between experimental groups. Metabolites with an FDR-adjusted p-value < 0.05 and log2fold change >1 were considered significantly differentially regulated between conditions. Metabolites identified as significantly altered were subsequently subjected to pathway enrichment analysis using the Functional Analysis module in MetaboAnalyst. Statistical significance for pathway enrichment was determined using an FDR-corrected threshold of p < 0.05.

### 2.9 Glucose and lactate measurement

Glucose and lactate concentrations were quantified in medium collected on days 1, 3, 5, and 7. Glucose concentration was measured using a commercial colorimetric Glucose Assay Kit (GAGO-20, Sigma–Aldrich) according to the manufacturer's instructions. Briefly, 50 μL of sample was mixed with 50 μL of glucose oxidase/peroxidase reagent containing *o*-dianisidine and incubated for 30 min at 37°C. The reaction was stopped by adding 50 μL of 6 M sulfuric acid, and absorbance was measured at 540 nm. Glucose concentrations were calculated from a standard curve. Lactate concentration was measured using a commercial Lactate Assay Kit (MAK064, Sigma–Aldrich) according to the manufacturer's instructions. Briefly, 12.5 μL of sample was mixed with 12.5 μL of assay buffer and 12.5 μL of Master Reaction Mix in a 96-well plate. Lactate concentration was determined at 570 nm using a standard curve generated according to the manufacturer's protocol.

### 2.10 Statistical analysis

Quantitative data are presented as mean ± standard deviation (SD). Statistical analyses were performed using GraphPad Prism 10. Differences between groups were assessed using two-way ANOVA followed by Tukey's post hoc multiple comparison test, unless otherwise specified in the figure legends. Glucose and lactate measurements were analyzed using linear mixed-effects models with glucose level, oxygen tension, day, and their interactions as fixed effects and donor as a random effect in R version 4.6.1 (lme4, lmerTest). Pairwise comparisons between conditions within each day were performed using Tukey correction. A p-value < 0.05 was considered statistically significant.

## 3 Results

### 3.1 Cell viability

Cell viability in cartilage, subchondral bone, and synovium tissues was assessed separately after 7 days of co-culture in either HG or LG medium and under two oxygen tensions (21% O_2_, considered hyperoxic; 5% O_2_, considered physioxic).

In cartilage, cell viability was assessed using LDH/ethidium homodimer staining. Viability varied across the cartilage depth and between the central and peripheral regions of the explants. In the peripheral region, cells were predominantly viable; therefore, quantitative evaluation of cell viability was focused on the central region of the explant, where the greatest variability was observed microscopically (Fig. 2A). In the region close to the articular surface (∼0–550 μm), no differences in the cell viability were observed between different glucose or oxygen conditions. This pattern persisted through the∼550–880 μm and ∼880-1100 μm zones, where LG condition showed a trend toward more dead cells compared to HG condition, although this difference did not reach significance. This effect became significant in the deeper area (∼1100–1400 μm), where LG resulted in higher cell death in both oxygen conditions (81 and 121 dead cells/area, in LG-21% and LG-5% O_2_ vs. 6 and 9 dead cells/area, in HG-21% and HG-5% O_2_; *p* < 0.05 and *p* < 0.001, respectively). In the deepest area (∼1400–1600 μm, present only in thicker samples), cell death remained to be higher under LG than HG in both oxygen levels, although there was no significant difference due to donor variability (Fig. 2B). Since the cartilage thickness varied across donors and conditions, the deep zone associated with the highest LG-induced cell death was only detected in those with thickness higher than about 1500 μm. Under HG, cell viability remained high (>95%) across the full thickness regardless of sample thickness (Fig. 2C). Under LG condition, however, cell viability was variable dependent on cartilage thickness with higher viability in thinner cartilage samples (≤1500 μm, 90-96%) and lower viability in thicker samples (>1500 μm, 37–46% under 5% O_2_; 54–64% under 21% O_2_). Reduced cell viability under LG condition reached significancy under 5% O_2_ (*p* < 0.05).

In subchondral bone, LDH/ethidium homodimer staining also indicated greater cell viability at the explant periphery than in the central region. In the central region, cell viability was higher in HG than LG, reaching significance only under 5% O_2_ (50% vs. 18% viability, *p* < 0.05; Fig. 2E). At the explant edge, LG significantly reduced cell viability compared with HG under both oxygen conditions (LG: 38% at 21% O_2_ and 33% at 5% O_2_; HG: 70% at 21% O_2_ and 65% at 5% O_2_; *p* < 0.05; Fig. 2F).

In synovium tissue, calcein/ethidium homodimer staining showed that synovial cell viability was not affected by glucose or oxygen levels. Cell viability remained high across all conditions: 88% (LG-21% O_2_), 85% (LG-5% O_2_), 87% (HG-21% O_2_), and 81% (HG-5% O_2_; Fig. 2G and H).

### 3.2 GAG and NO release from whole-joint tissues

GAG and NO levels in conditioned media were quantified over the 7 days culture period (Fig. 3A and B, cumulative release). The release of GAG and NO was not significantly influenced by glucose concentration or oxygen tension. Qualitative assessment of Safranin O/Fast Green stained histological sections did not reveal obvious differences in proteoglycan expression between culture conditions (Fig. 3C).

**Fig. 3:**
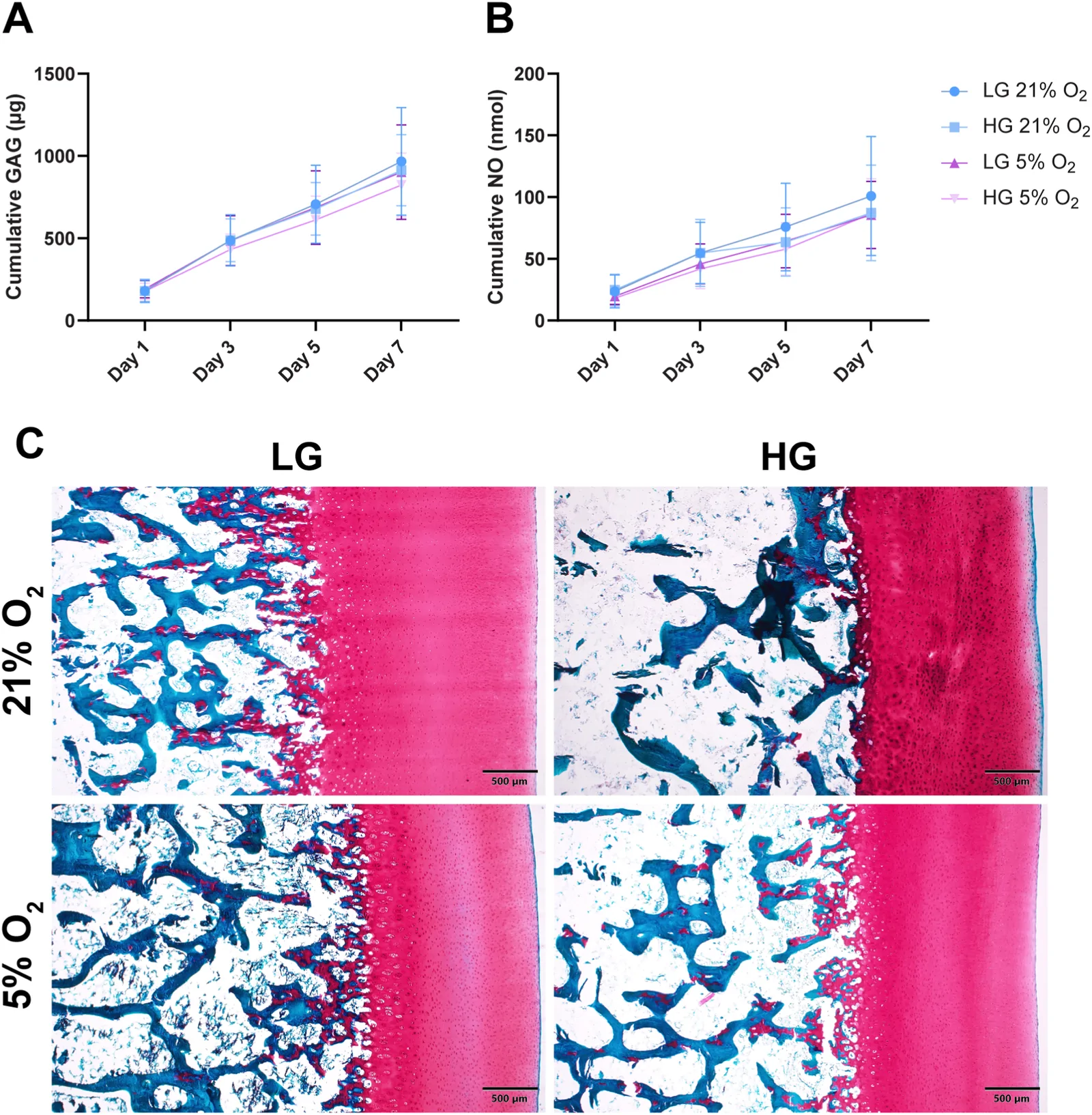
GAG and NO release. Cumulative release of (A) GAG and (B) NO during 7 days of culture in the presence of low glucose (LG) and high glucose (HG) DMEM under physioxia (5% O_2_) and hyperoxia (21% O_2_) conditions. (C) Safranin O/Fast Green staining of osteochondral tissues after 7 days of culture. Results of GAG and NO release are presented as means of 4 replicates from 5 donors for every tissue (n = 20). Statistical analysis was performed using 2-way ANOVA.

### 3.3 Gene expression in individual joint tissues: cartilage, bone, and synovium

RT-qPCR analysis was performed separately on cartilage, subchondral bone, and synovium after 7 days of co-culture for different experimental conditions. Respective tissues on day 0 served as controls for all analyses.

In cartilage, *ACAN* transcript levels were upregulated in HG compared with LG under 21% O_2_ (p < 0.05), whereas no difference was detected under 5% O_2_ (Fig. 4A). *COL2* and *COMP* expressions were reduced in LG under 21% O_2_ compared with LG under 5% O_2_; however, these transcripts were not affected by glucose concentration (Fig. 4B and C, respectively). *COL10*, *ADAMTS4*, *MMP13*, and *IL6* transcripts were all upregulated in HG compared with LG under 21% O_2_ (p < 0.01, 0.001, 0.01, and 0.05, respectively) (Fig. 4D, E, 4F and 4G, respectively). These differences were not observed under 5% O_2_, as transcript levels in HG were significantly lower at 5% O_2_ than at 21% O_2_ (p < 0.0001, 0.05, 0.0001, and 0.001 for *COL10*, *ADAMTS4*, *MMP13*, and *IL6*, respectively). *IL8* expression was not affected by either glucose or oxygen concentration (Fig. 4H).

**Fig. 4:**
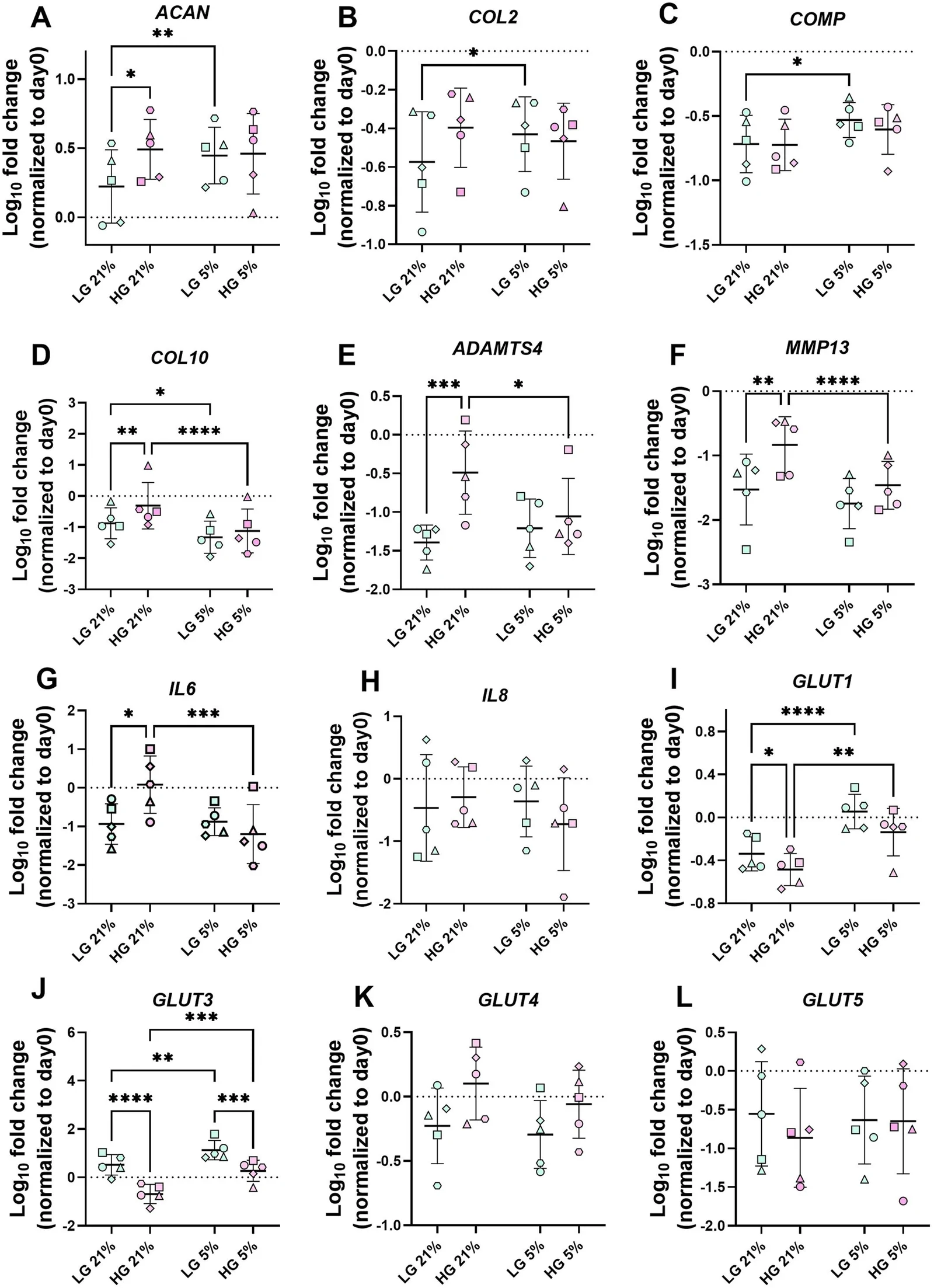
Gene expression levels in cartilage tissue cultured for 7 days in low glucose (LG) and high glucose (HG) DMEM under physioxia (5% O_2_) and hyperoxia (21% O_2_) conditions: (A) *ACAN*, (B) *COL2*, (C) *COMP*, (D) *COL10*, (E) *ADAMTS4*, (F) *MMP13*, (G) *IL6*, (H) *IL8*, (I) *GLUT1*, (J) *GLUT3*, (K) *GLUT4*, and (L) *GLUT5*. Data is normalized to fresh tissue collected on day 0. Results are presented as means of three replicates per donor (n = 5 donors, depicted with different symbols for each donor). Statistical analysis was performed using 2-way ANOVA, with significance levels indicated as *p < 0.05, **p < 0.01, ***p < 0.001, ****p < 0.0001.

Expression of glucose transporters *GLUT1*, *GLUT2*, *GLUT3*, *GLUT4*, and *GLUT5* was also evaluated. *GLUT2* transcripts were not detected in cartilage. *GLUT1* and *GLUT3* were upregulated in LG compared with HG under both oxygen conditions (Fig. 4I and J respectively); for *GLUT1*, this difference did not reach significance at 5% O_2_. Exposure to 5% O_2_ increased the expression of both transporters in LG and HG (*GLUT1*: p < 0.0001 and 0.001; *GLUT3*: p < 0.01 and 0.001 in LG and HG, respectively). *GLUT4* and *GLUT5* transcript levels were not affected by glucose or oxygen concentration (Fig. 4K and L, respectively).

In subchondral bone, *ALPL* transcript levels were downregulated under 5% O_2_ compared with 21% O_2_ in both LG and HG conditions (p < 0.05 and p < 0.001, respectively; Fig. 5A). *COL1* and *IL8* expressions were not affected by either glucose or oxygen condition (Fig. 5B and E, respectively). *BGLAP* and *IL6* expression was insensitive to glucose levels but was reduced under 21% O_2_ compared with 5% O_2_ (p < 0.01 and p < 0.05, respectively; Fig. 5C and F, respectively). *VEGF* transcript levels were upregulated in LG compared with HG under 5% O_2_ (p < 0.01) and were also elevated under 5% O_2_ compared with 21% O_2_ within the LG condition (p < 0.05; Fig. 5D). Analysis of glucose transporter gene expression revealed that *GLUT1* was downregulated in HG compared with LG at both 21% and 5% O_2_ (p < 0.01, Fig. 5G). In addition, *GLUT1* expression increased in HG under 5% O_2_ relative to 21% O_2_ (p < 0.05). *GLUT3* was downregulated in HG compared with LG only under 5% O_2_ (p < 0.01, Fig. 5H). *GLUT4* was upregulated in HG relative to LG under 21% O_2_, and this increase was diminished under 5% O_2_ (p < 0.001, Fig. 5I). *GLUT5* expression was not influenced by glucose concentration or oxygen tension (Fig. 5J).

**Fig. 5:**
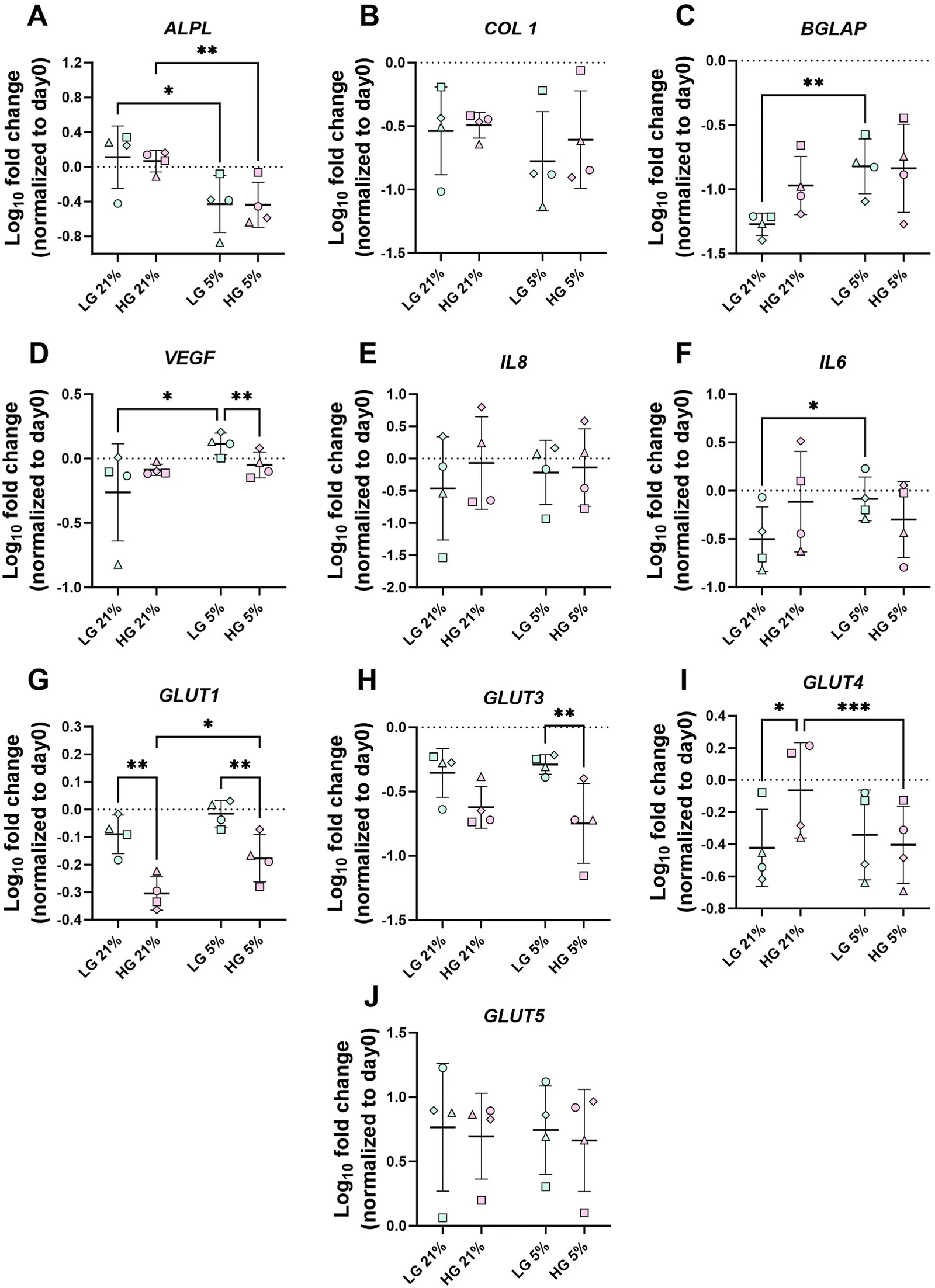
Gene expression levels in subchondral bone tissue cultured for 7 days in low glucose (LG) and high glucose (HG) DMEM under physioxia (5% O_2_) and hyperoxia (21% O_2_) conditions: (A) *ALPL*, (B) *COL1*, (C) *BGLAP*, (D) *VEGF*, (E) *IL8*, (F) *IL6*, (G) *GLUT1*, (H) *GLUT3*, (I) *GLUT4*, and (J) *GLUT5*. Data is normalized to fresh tissue collected on day 0. Results are presented as means of three replicates per donor (n = 4 donors, depicted with different symbols for each donor). Statistical analysis was performed using 2-way ANOVA, with significance levels indicated as *p < 0.05, **p < 0.01, ***p < 0.001, ****p < 0.0001.

In synovium, *VEGF*, *MMP3*, and *IL8* transcript levels were not affected by glucose or oxygen condition (Fig. 6A, C, and 6F, respectively). *ADAMTS5* and *MMP1* showed differential sensitivity to oxygen: *ADAMTS5* was downregulated in LG condition compared to HG under both 21% and 5% O_2_ (p < 0.01 in 21% O_2_; p < 0.001 in 5% O_2_ Fig. 6B), whereas *MMP1* was downregulated under 5% O_2_ in both glucose conditions (p < 0.05, Fig. 6D, respectively). *IL6* expression was increased in HG compared with LG, but only under 21% O_2_ (p < 0.0001, Fig. 6E). Analysis of glucose transporters showed that *GLUT1* expression was unaffected by glucose or oxygen (Fig. 6G), and *GLUT2* was not detectable in synovium. *GLUT3* expression was reduced in HG compared with LG under 21% O_2_ (p < 0.05, Fig. 6H), and this difference was diminished under 5% O_2_. *GLUT4* expression was higher in HG than LG under 21% O_2_ (p < 0.05, Fig. 6I), with no differences observed at 5% O_2_. *GLUT5* expression was decreased under 5% O_2_ compared with 21% O_2_, but only in LG (p < 0.05, Fig. 6J).

**Fig. 6:**
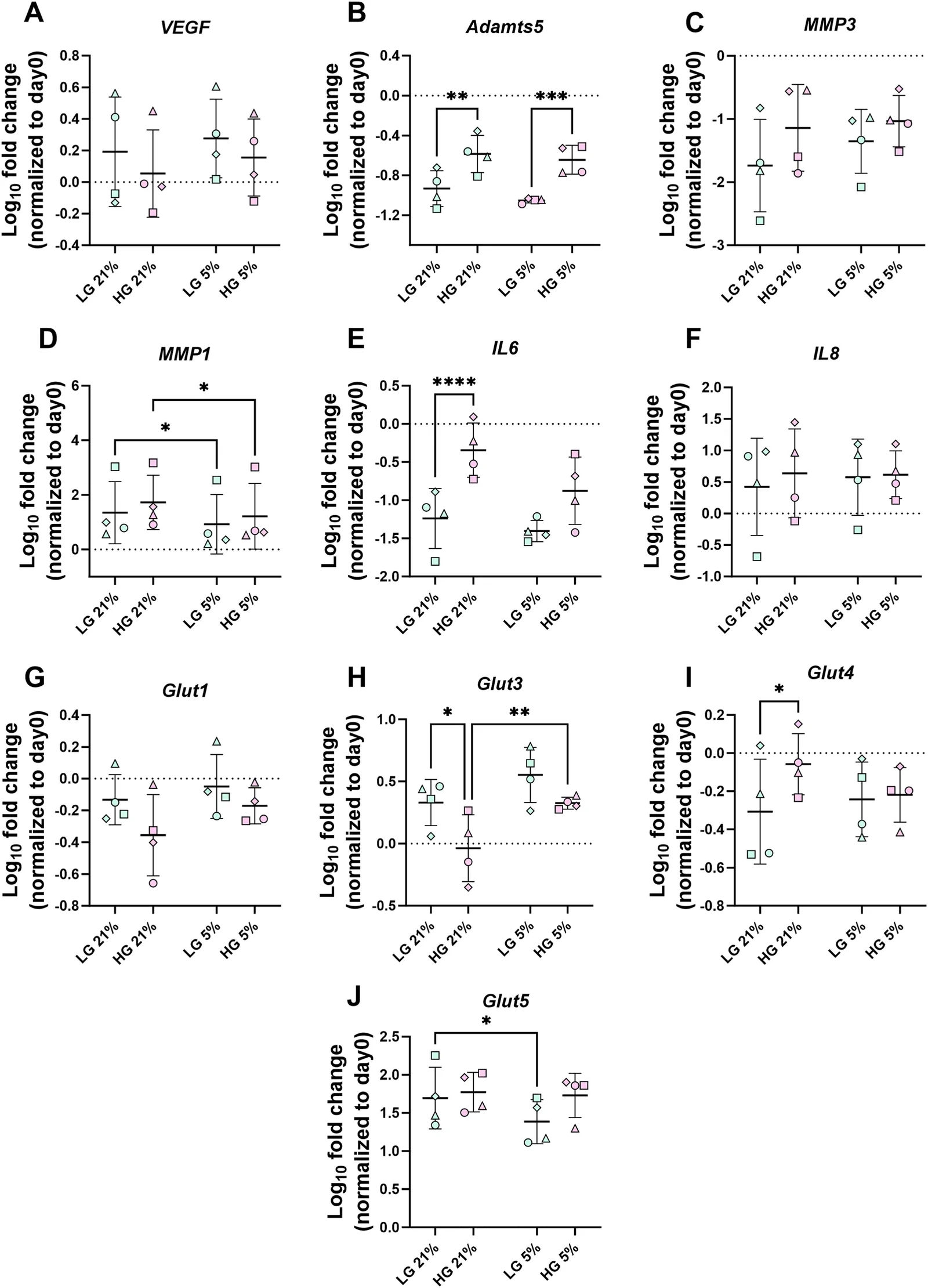
Gene expression levels in synovium tissue cultured for 7 days in low glucose (LG) and high glucose (HG) DMEM under physioxia (5% O_2_) and hyperoxia (21% O_2_) conditions: (A) *VEGF*, (B) *ADAMTS5*, (C) *MMP3*, (D) *MMP1*, (E) *IL6*, (F) *IL8*, (G) *GLUT1*, (H) *GLUT3*, (I) *GLUT4*, and (J) *GLUT5*. Data is normalized to fresh tissue collected on day 0. Results are presented as means of three replicates per donor (n = 4 donors, depicted with different symbols for each donor). Statistical analysis was performed using 2-way ANOVA, with significance levels indicated as *p < 0.05, **p < 0.01, ***p < 0.001, ****p < 0.0001.

### 3.4 Metabolomics in individual joint tissues: cartilage, bone, and synovium

To examine the metabolomic profiles of tissues cultured under varying glucose and oxygen levels, we performed untargeted metabolomics on cartilage, subchondral bone, and synovium separately. Fresh tissue from each compartment was processed in parallel and used as a control. In total, 121 metabolites were detected across cartilage, bone, and synovium.

Principal component analysis (PCA) revealed a clear separation between fresh tissues and cultured tissues for 7 days (Fig. 7A, B, C), a pattern observed consistently across all three tissue types. To evaluate differences associated with the culture variables themselves, PCA was subsequently performed on all cultured samples without the control group (Supplementary material, Fig. S1). To evaluate the effects of glucose and oxygen levels specifically, PCA was then repeated for each of the two conditions, separately. In cartilage, under 21% O_2_, HG and LG samples showed modest separation with partial overlap, whereas under 5% O_2_ no clustering between the two glucose conditions was observed. When comparing oxygen levels, a slight distinction between 21% and 5% O_2_ was evident in the HG groups (PC1: 39.1%), while LG samples showed no clear separation (Fig. 7A). In subchondral bone, HG and LG conditions showed a clear separation under 21% O_2_, primarily along PC2 (20.5%). This clustering was not observed under 5% O_2_. Comparison of oxygen levels revealed a strong separation between 21% and 5% O_2_ within the LG groups along PC2 (23.3%), whereas HG samples exhibited only a weak distinction (Fig. 7B). Synovial tissue showed the strongest glucose-driven effects. HG and LG samples formed distinct clusters under both oxygen levels, with separation occurring mainly along PC1 (42.8% at 21% O_2_; 38.5% at 5% O_2_). When comparing oxygen levels, HG samples showed only slight clustering between 21% and 5% O_2_ (PC1: 36%), while LG samples demonstrated clearer separation (PC1: 36.8%, Fig. 7C).

**Fig. 7:**
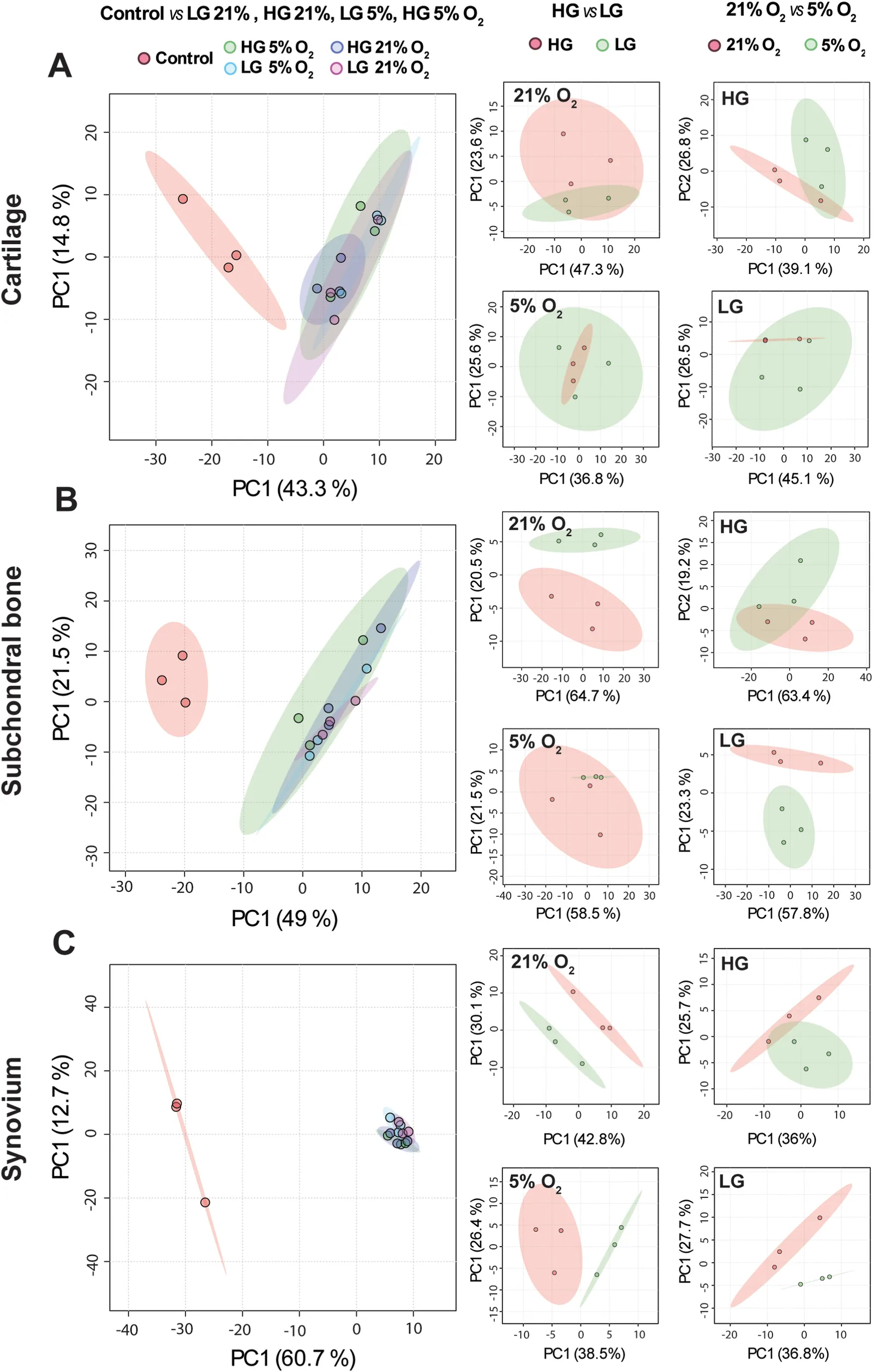
Principal component analysis (PCA) of metabolomics data of (A) cartilage, (B) bone and (C) synovium cultured for 7 days in LG or HG medium under 21% or 5% O_2_ concentration. Fresh tissue is considered as control for each tissue type.

Evaluation of differentially expressed metabolites (log_2_ fold change [log_2_FC] > 1) revealed only a limited number of metabolites, with 5–7 in cartilage, 8–14 in bone, and 7–11 in synovium showing significant changes across conditions (supplementary material, Tables S1–S3). Among the altered metabolites in cartilage, comparison of HG and LG conditions under 21% O_2_ and 5% O_2_ showed elevated levels of glucose, glucosamine, gluconic acid, while uracil and cAMP were decreased in HG compared to LG (Table S1). Under HG and LG conditions, hypoxanthine and ethyl sulfate were increased whereas 4-pyridoxic acid was decreased in 5% O_2_ compared with 21% O_2_ (Table S1). Among the altered metabolites in bone and synovium, comparison of HG and LG conditions under 21% O_2_ and 5% O_2_ showed increased glucose and gluconic acid levels, and decreased uracil in HG similar to what was observed in cartilage (Tables S2 and S3). Under both HG and LG conditions, 5% O_2_ downregulated 4-pyridoxic acid, adenine, adenosine, and guanosine, compared with 21% O_2_, whereas ethyl sulfate was upregulated (Tables S2 and S3).

Pathway analysis of all altered metabolites identified several pathways, mostly represented by a single metabolite, across the experimental comparisons, including 21% and 5% O_2_, as well as the three tissue types (synovium, subchondral bone, and cartilage). However, none of the pathways reached statistical significance after FDR correction. Therefore, the results of pathway analysis should be considered exploratory and hypothesis-generating rather than evidence of pathway regulation. Across both glucose conditions, pathway enrichment analysis suggested potential involvement of purine metabolism, vitamin B6 metabolism, and the biosynthesis of multiple amino acids (valine, leucine, isoleucine, arginine, pyrimidine, alanine, tryptophan, cysteine, aspartate, and glutamate) in the response to oxygen availability across all three tissues (Fig. S2A–C).

Assessment of glucose levels in cartilage, bone, and synovium, both in fresh control tissues and after 7 days of the culture revealed distinct differences among groups. In cartilage and bone, control samples displayed a broader distribution of normalized log2-transformed glucose levels. Across both tissues, clear differences were observed between HG and LG conditions, with LG groups (at both 21% and 5% O_2_) consistently exhibiting lower glucose levels although it was not significant (p > 0.5). In synovium, glucose levels in the control group were comparable to those observed in samples cultured with LG medium under 5% O_2_ (Fig. S3).

To further evaluate tissue metabolic activity under the different culture conditions, glucose consumption and lactate production (Fig. 8A and B) were quantified in the medium on days 1, 3, 5, and 7 (n = 3). Glucose consumption was higher in HG than LG cultures on day 3 under both 21% O_2_ (8 vs. 3 mg, *p* < 0.001) and 5% O_2_ (6.3 vs. 3.1 mg, *p* < 0.001). While this difference remained significant under 21% O_2_ on days 5 and 7 (*p* < 0.001 and *p* < 0.05, respectively), it was attenuated under 5% O_2_ on day 5 (*p* < 0.05) and was no longer significant on day 7 (*p >* 0.05*).* No differences were detected between 21% and 5% O_2_ maintained at LG cultures at any time point. Lactate production was also higher in HG compared to LG cultures from day 3 onwards under both 21% O_2_ (*p* < 0.001) and 5% O_2_ (day 3: *p* < 0.01; day 5: *p* < 0.05, day 7: *p* < 0.001). However, lactate production was reduced under 5% O_2_ compared with 21% O_2_ in HG cultures on days 5 and 7 (*p* < 0.01 and *p* < 0.001, respectively), whereas no differences in lactate production were detected between 21% and 5% O_2_ maintained at LG cultures at any time point.

**Fig. 8:**
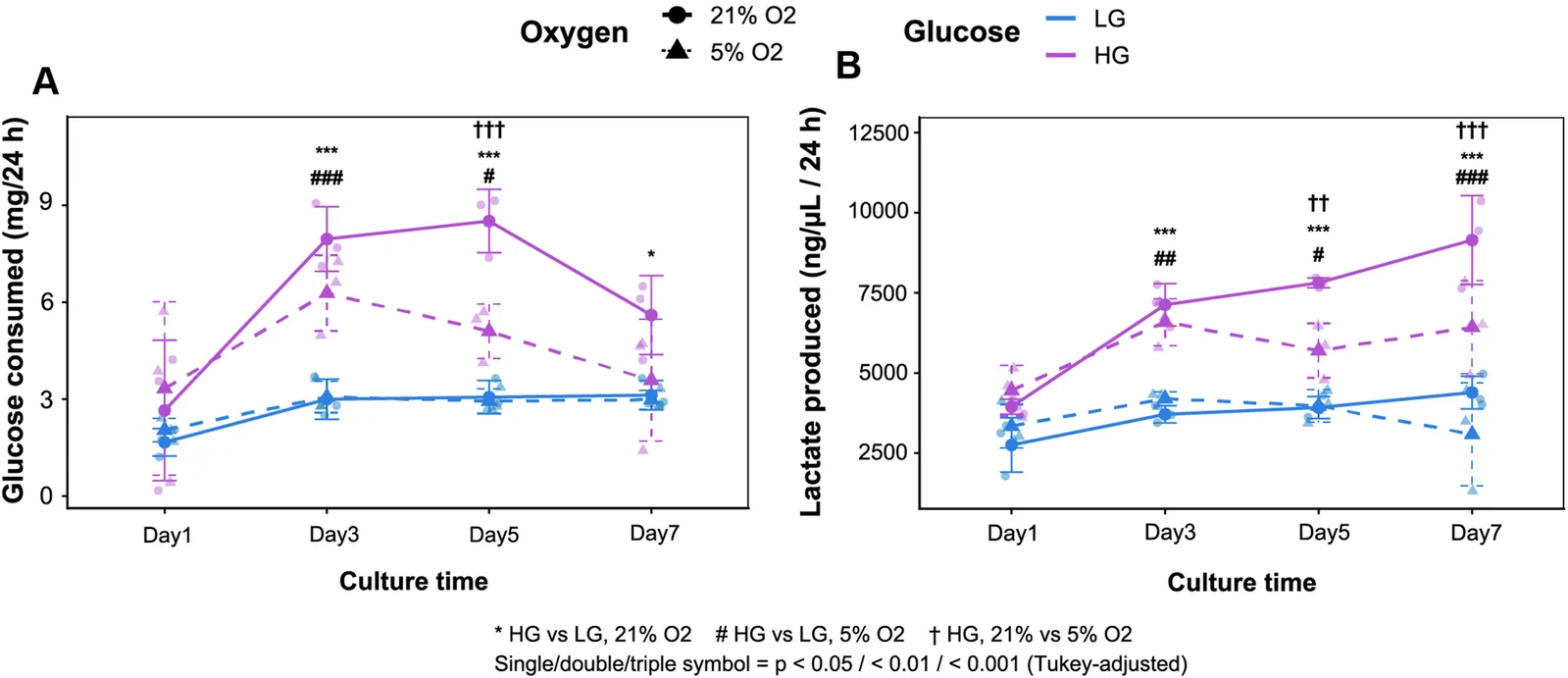
**Glucose consumption and lactate production under different glucose and oxygen conditions.** (A) Glucose consumption and (B) lactate production were quantified in conditioned medium collected on days 1, 3, 5, and 7 from osteochondral-synovial co-cultures maintained in low glucose (LG, 1 g/L) or high glucose (HG, 4.5 g/L) medium under hyperoxic (21% O_2_) or physioxic (5% O_2_) conditions. Values are presented as mean ± SD (n = 3). Statistical analysis was performed using linear mixed-effects models with Tukey-adjusted post hoc tests (donor included as a random effect).

## 4 Discussion

One of the most important factors in a successful tissue culture is to maintain cell viability. Investigating cell viability in cartilage, bone and synovium revealed an increase of cell death in cartilage and subchondral bone under LG culture condition independent of oxygen level. This was not observed in synovium and may be explained by its low thickness which allows a more uniform nutrient diffusion across the tissue. However, in cartilage and bone, the high number of dead cells in the central parts of the tissue which are less accessible to nutrients due to diffusion-consumption limitations, suggests that LG medium fails to support cell maintenance and hence their viability in the entire explant. These results are consistent with a report showing that high glucose level increases cell viability in cartilage [29]. The failure of LG medium to maintain cell viability in deeper regions of cartilage and bone suggests that physiological glucose concentrations alone are insufficient to meet the high metabolic demands of these tissues *ex vivo*. A few factors are likely to contribute to this observation. First, the lack of blood supply in *ex vivo* subchondral bone cultures restricts nutrient transport, leaving cells reliant solely on diffusion from the surrounding medium. Second, static culture conditions fail to replicate *in vivo* dynamics, where synovial fluid is replenished approximately every 2 h [30]. Although we replaced the medium daily, this cannot fully mimic *in vivo* nutrient supply. Consistent with this, glucose consumption measurements indicated that a large proportion (about 84%) of the glucose in LG medium was depleted between media changes, whereas substantial amounts of glucose (about 69%) remained available in HG medium. Consequently, in HG medium the higher glucose concentration maintained a greater concentration gradient for diffusion into the tissue, thereby supporting a higher glucose flux and better sustaining cellular metabolic needs and viability throughout static culture, despite exceeding physiological glucose levels. However, it should be acknowledged that static culture does not reproduce the dynamic conditions present *in vivo*, which may also affect fluid movement and nutrient transport. Although cyclic loading may induce interstitial fluid flow, O'Hara et al. found no significant enhancement of small solute transport through articular cartilage, indicating that glucose and oxygen transport remain predominantly diffusion driven [31]. Convection induced by loading may be more relevant for larger, less diffusible solutes [32]. However, medium flow may improve nutrient exchange within osteochondral constructs, particularly in the subchondral bone compartment. Consistent with this, rotary shaker culture improved viability in the middle and deep cartilage zones and reduced apoptosis in the subchondral bone compared with conventional static culture [33]. Thus, the reduced viability under LG conditions likely reflects limited nutrient availability within this static culture setup. On the other hand, even in physiologically loaded bovine intervertebral disc tissue (0.02–0.20 MPa, 0.2 Hz, 2 h/day for 7 days), reducing glucose from 25 to 5.5 mM markedly decreased cell viability, indicating that mechanical loading did not overcome glucose limitation [34].

In the present study, in cartilage, the expression of key anabolic gene *ACAN* was reduced by LG under 21% O_2_, likely reflecting the critical role of glucose as a precursor for glycoprotein and glycosaminoglycan synthesis necessary for extracellular matrix formation. However, the expression of *ACAN*, as well as *COL2* and *COMP* was induced under physiologically relevant oxygen condition (5% O_2_), indicating that oxygen availability influences the regulation of cartilage anabolic genes. This finding aligns with previous studies showing that low oxygen conditions (5% O_2_) enhance the expression of *SOX9* and *ACAN* in human chondrocytes compared to 21% O_2_ [35] supporting a physioxia-driven anabolic response in cartilage. These transcriptional changes were not reflected in GAG or NO release or in histological matrix staining, suggesting that the observed gene expression responses did not lead to measurable alterations in extracellular matrix composition during the culture period.

Additionally, HG conditions increased the expression of several catabolic, hypertrophic, and inflammatory genes in cartilage, including *ADAMTS4*, *MMP13*, *COL10*, and *IL6*, specifically under 21% of O_2_. Notably, these glucose-dependent differences were not observed under 5% O_2_, where gene expression levels under LG and HG conditions became comparable. Consistent with this, metabolomics analysis showed that LG and HG groups exhibited more similar metabolite profiles at 5% O_2_ than at 21% O_2_, indicating that physioxia buffers or overrides glucose-mediated metabolic responses. It has been previously reported that both high or deprived glucose levels, as well as oxygen deprivation (1% O_2_) can independently exert pro-inflammatory effects and induce IL6 expression [[36], [37], [38], [39]]. However, 5% O_2_ has also been reported to counteract glucose-deprivation–induced IL6 expression in microglia, macrophages, and monocytes compared with 21% O_2_ [40]. This antagonistic effect appears to occur through Akt signaling rather than HIF-1/2α pathway. Together with our findings, this supports the presence of a crosstalk between glucose availability and physioxia pathways, suggesting that transcriptional and metabolic responses to glucose interact with oxygen-sensitive pathways.

In bone and synovium, the effects of glucose and oxygen on gene expression were gene specific. For instance, *ALPL* expression in bone was highly regulated by oxygen but insensitive to glucose availability. This is consistent with multiple studies demonstrating that low oxygen (2–5%) reduces ALPL mRNA, its enzymatic activity, and the formation of mineralized bone nodules [[41], [42], [43]]. *BGLAP*, which encodes osteocalcin, showed a pattern similar to anabolic genes in cartilage: it was downregulated by LG at 21% O_2_ but unaffected by LG under 5% O_2_. Osteocalcin is a bone-derived endocrine hormone that regulates systemic glucose homeostasis by stimulating insulin secretion in pancreatic cells and increasing peripheral insulin sensitivity. Hyperglycemia in type 2 Diabetes Mellitus Patients has been shown to decrease osteocalcin levels in the blood [44]. Although this appears inconsistent with our *ex vivo* observation, the decreased osteocalcin in patients more likely reflects disturbed bone energy metabolism or impaired osteoblast function rather than a direct suppressive effect of glucose on *BGLAP* transcription. *VEGF* expression in bone but not in synovium, was modulated by oxygen. Under LG conditions, 5% O_2_ significantly increased *VEGF*, consistent with the classical HIF-1α–mediated stimulation of angiogenic genes observed under low oxygen conditions [45,46]. Moreover, previous studies show that low glucose availability results in intracellular ATP depletion which increases *VEGF* mRNA expression [47]. Thus, our result suggests that oxygen-dependent *VEGF* expression is further modulated by cellular energy status, with glucose availability shaping the magnitude of the response.

In synovium, *IL6* followed a pattern similar to cartilage, increasing under HG only at 21% O_2_. This suggests that cartilage and synovium share a comparable glucose–oxygen interaction profile for *IL6* regulation. However, catabolic markers *MMP1* and *ADAMTS5* displayed divergent responses. *ADAMTS5* expression was reduced by LG in both oxygen levels, whereas *MMP1* expression was decreased under both LG and HG at 5% O_2_. These different patterns indicate a more complex, tissue specific regulatory landscape in synovium, likely reflecting its heterogeneous cellular composition, including fibroblasts, macrophages, and endothelial cells, in contrast to the relatively homogeneous chondrocyte population in cartilage. Metabolomics further supported this complexity. Synovium showed the strongest metabolic response to glucose and oxygen, with clear PCA clustering between experimental conditions. This clustering was driven primarily by glucose concentration, while in bone the dominant driver was oxygen. Cartilage displayed the weakest clustering, suggesting a more constrained or buffered metabolic plasticity compared with synovium and bone. Owing to the hypoxic nature of cartilage tissue and its dependence on glycolysis, larger metabolic shifts are expected in bone and synovium under low glucose and oxygen availability.

Our data further indicates that glucose and oxygen levels jointly regulate the expression of specific glucose transporters in a tissue-dependent manner. In cartilage, *GLUT1* and *GLUT3* were responsive to both glucose concentration and oxygen levels; in bone, *GLUT1* and *GLUT4* showed similar regulation; and in synovium, *GLUT3* and *GLUT5* were affected. Overall, LG and 5% O_2_ tended to increase *GLUT1* and *GLUT3*, whereas HG and 21% O_2_ increased *GLUT4*, and LG combined with 5% O_2_ decreased *GLUT5* expression. Notably, analysis of baseline transporter expression in native tissues demonstrated that *GLUT1* and *GLUT3* were generally expressed at higher levels than *GLUT4* and *GLUT5*, whereas *GLUT5* showed the lowest expression across most tissues (Table S4). Glucose is the primary energy source for mammalian cells, and its availability can fluctuate over time. Cells adapt to such fluctuations through several mechanisms, one of the most important being the modulation of glucose transporter expression. Enhanced *GLUT1* and *GLUT3* expressions in different cell types under low oxygen and through HIF-1α signaling is well known [48]. In agreement with our findings in cartilage, *GLUT1* expression in chondrocytes has been reported to increase under hypoxic or glucose-deprived conditions and to decrease under high glucose conditions, consistent with its adaptive role in maintaining cellular glucose homeostasis [49]. Furthermore, low oxygen and glucose deprivation act synergistically to upregulate *GLUT1*, *GLUT3*, and *VEGF* expression in pancreatic cancer cells [50]. *GLUT1*, *GLUT3*, and *GLUT4* exhibit high affinity for glucose, while *GLUT5* has a high affinity for fructose. In our study, *GLUT5* expression was low in native tissues compared to the other glucose transporters, but its expression increased after *ex vivo* culture. This likely reflects adaptations to altered nutrient composition in the culture medium, which can shift cellular metabolism. This is supported by the observed substantial differences in metabolite profiles between native tissues and tissues cultured for 7 days, highlighting the metabolic remodeling induced by *ex vivo* conditions.

Metabolomics analysis revealed oxygen dependent modulation of vitamin B6 metabolism, characterized by consistently reduced levels of 4-pyridoxic acid across cartilage, bone, and synovium under 5% O_2_ compared with 21% O_2_. 4-Pyridoxic acid is a catabolic product of vitamin B6 turnover. The active form of vitamin B6 is Pyridoxal 5′-phosphate (PLP). It is a coenzyme for many amino acid–metabolizing enzymes and is produced from pyridoxine, which is present in DMEM in the form of pyridoxine hydrochloride, via sequential phosphorylation by pyridoxal kinase and oxidation by pyridoxine 5′-phosphate oxidase (PNPO) [51,52]. The observed reduction in 4-pyridoxic acid indicates diminished degradation of PLP. PNPO has been identified as an oxygen-sensing factor that suppresses lysosomal activity in macrophages under prolonged hypoxia (1% O_2_), independent of the HIF pathway [53]. The reduction in 4-pyridoxic acid observed under physioxia may therefore suggest oxygen-dependent regulation of vitamin B6 turnover. Furthermore, hypoxic cells (<1% O_2_) rely heavily on amino acid metabolism [54,55], as reflected by decreased levels of metabolites associated with various amino acid pathways in subchondral bone in our data. Although 5% O_2_ is considered physioxic for cartilage, this oxygen level may represent a relative reduction in oxygen availability for subchondral bone due to its vascularized nature *in vivo.* This metabolic shift increases demand for PLP-dependent transamination reactions, reinforcing the potential cellular strategy of reducing vitamin B6 breakdown to maintain sufficient PLP levels.

Across all joint tissues, several purine-related metabolites were modulated by oxygen availability. In cartilage, physioxia-induced accumulation of hypoxanthine is consistent with previous reports [56] demonstrating that reduced oxygen availability alters purine metabolism and increases the breakdown of adenine nucleotides to meet cellular energy demands [[57], [58], [59]]. In synovium and bone, by contrast, reduced levels of purine nucleotides and bases (adenosine, guanosine, and adenine) as well as allantoin, the end product of the purine catabolism in mammals, reflect tissue-specific regulation of purine metabolism under physioxic conditions.

Although reduced oxygen availability is often associated with a shift in cellular metabolism toward enhanced glycolysis, previous studies have demonstrated increased glycolysis and lactate production under hyperglycemic conditions [60]. Consistent with this, HG cultures in the present study exhibited greater glucose consumption and lactate accumulation than LG cultures, indicating that glucose availability was a major determinant of glycolytic activity. Under physioxia, however, glucose-dependent increases in glucose consumption and lactate production were attenuated compared with hyperoxia, resulting in more similar metabolic responses between HG and LG cultures. This result is consistent with early work in bovine articular cartilage, showing that lactate production decreases with reduced oxygen availability [61]. Moreover, while the observed purine-related metabolite changes may suggest local adaptations to oxygen availability within individual tissues, the overall metabolic activity of the co-culture system appeared to be more strongly influenced by glucose availability.

Our findings also revealed a distinct tissue specific pattern of glucose concentration. Comparison of glucose content in fresh tissues with explants cultured for seven days showed that fresh synovium contained glucose levels similar to those cultured under LG culture conditions. This was expected, since LG concentration is the physiological concentration of glucose in synovial fluid similar to blood. In contrast, fresh cartilage and subchondral bone tissues showed glucose levels more comparable to explants maintained in HG medium, although this pattern displayed donor-to-donor variability. This might indicate that HG medium can help maintain glucose levels in cartilage and bone at a level similar to *in vivo* conditions, however these results should be further confirmed using a larger number of donors to better define the native glucose profile of cartilage and subchondral bone.

This study has several limitations that should be considered when interpreting the results and addressed in future studies. First, the number of biological donors was relatively limited (n = 3-5) which may restrict the generalizability of the findings. Therefore, future studies, including a larger donor cohort, are required to further validate these results. Second, all tissues were cultured in the same medium, which does not fully reflect the *in vivo* joint environment. While cartilage and synovium are exposed to synovial fluid, subchondral bone receives nutrients from the vascular system and is not directly exposed to synovial fluid. Future studies employing compartmentalized culture systems may provide a more physiologically relevant model while preserving tissue crosstalk. Third, a single oxygen tension (5% O_2_) was applied to all tissues despite the fact that cartilage, synovium, and subchondral bone experience different oxygen environments *in vivo*. Future studies incorporating tissue-specific oxygen conditions may better reflect the native oxygen microenvironment of individual joint tissues and improve the translational relevance of the model. Additionally, tissue responses were evaluated only over a 7-day culture period. Although this duration was sufficient to identify early cellular, transcriptional, and metabolic responses to glucose and oxygen availability, longer culture periods may be required to evaluate long-term tissue adaptation to the microenvironment. Finally, species-specific differences limit the direct extrapolation of findings from bovine tissues to human physiology and clinical outcomes, requiring additional validation in human models.

## 5 Conclusion

Taken together, this study demonstrates that glucose concentration and oxygen availability modulate the homeostasis and metabolism of joint tissues in distinct, tissue-specific ways. High glucose medium appears most suitable for maintaining cartilage and subchondral bone viability under the current tested conditions, whereas synovium exhibited greater sensitivity to variations in its metabolic environment. The differential responses of synovium compared with cartilage and bone highlight the complexity of the joint microenvironment. This emphasizes the importance of carefully defining culture parameters in complex co-culture models. Nevertheless, culturing tissues under physioxic conditions (5% O_2_) helped buffer cartilage and bone responses to nutrient fluctuations, supporting a more stable and physiologically relevant metabolic state. Establishing such functional conditions is essential for developing clinically meaningful *ex vivo* platforms that can reduce reliance on animal models and accelerate translational research on OA and other joint diseases.

## Author contribution

F. Safari: Conceptualization, Methodology, Investigation, Data curation, Formal analysis, Writing – original draft, Writing – review & editing. J. Zvicer: Investigation, Data curation, Writing – original draft, Writing – review & editing. S. Grad: Conceptualization, Formal analysis, Writing – review & editing. M. J. Stoddart: Conceptualization, Formal analysis, Writing – review & editing. Z. Li: Conceptualization, Formal analysis, Supervision, Writing – review & editing.

## Ethics declaration

This study was conducted in accordance with the ARRIVE (Animal Research: Reporting of In Vivo Experiments) guidelines. Ethics committee approval was not required. Bovine stifle joints were purchased from a local abattoir. As the tissues were obtained from animals slaughtered for the food chain and not specifically for research purposes, ethical approval was not required.

## Data availability statement

The data that supports the metabolomics findings of this study are available from the corresponding author upon request.

## Declaration of generative Ai in scientific Writing

During the preparation of this work the authors used ChatGPT in order to polish the English grammar and spelling. After using this tool, the authors reviewed and edited the content as needed and took full responsibility for the content of the published article.

## Funding

This work was supported by the 10.13039/501100001702AO Foundation. Mobility and research exchange activities for Dr. Jovana Zvicer to visit the AO Research Institute were supported by the European Union’s Horizon 2020 research and innovation programme under grant agreement No. 952033 (ExcellMater – Twinning to Excel Materials Engineering for Medical Devices) and by the NetwOArk COST Action (CA21110), funded by 10.13039/501100000921COST (10.13039/501100000921European Cooperation in Science and Technology). Z. Li was funded by SINPAIN project, from European Union's Horizon Europe research and innovation programme under Grant Agreement NO. 101057778. The AO Research Institute Davos was funded by State Secretariat for Education, Research and Innovation (SERI) of Switzerland within the SINPAIN project. Views and opinions expressed are however those of the authors only and do not necessarily reflect those of the European Union. Neither the 10.13039/501100000780European Union nor the granting authority can be held responsible for them.

## **Declaration of competing interests**

The authors declare that they have no known competing financial interests or personal relationships that could have appeared to influence the work reported in this paper.
