# Supplementary material for: Establishing a functional osteochondral–synovial ex vivo joint model: Systematic evaluation of glucose and oxygen impact
Source: J Orthop Translat. 2026 Oct 24;61:101240. doi: 10.1016/j.jot.2026.101240 (PMC13721937; doi:10.1016/j.jot.2026.101240)
Supplement: Supplementary file 1 [file mmc1.docx]

**Table S1:** Differentially regulated metabolites in cartilage co-cultured with bone and synovium under 21% and 5% O_2_ and high glucose (HG) and low glucose (LG) medium for 7 days.

| **HG vs LG, 21% O_2_** | | | | |
| --- | --- | --- | --- | --- |
| **Metabolites** | **FC** | **log2(FC)** | **raw.pval** | **-LOG10(p)** |
| Uracil | 0.24091 | -2.0534 | 0.024236 | 1.6155 |
| THREONINE | 0.25692 | -1.9606 | 0.060481 | 1.2184 |
| UDP-N-acetylglucosamine | 2.0025 | 1.0018 | 0.087007 | 1.0604 |
| N-ACETYLSERINE | 2.4905 | 1.3164 | 0.032678 | 1.4858 |
| Glucuronic acid | 4.4036 | 2.1387 | 0.039211 | 1.4066 |
| Gluconic acid | 8.4046 | 3.0712 | 0.000438 | 3.3587 |
| Glucose | 9.2894 | 3.2156 | 0.00414 | 2.383 |
| **HG vs LG, 5% O_2_** | | | | |
| cAMP | 0.23195 | -2.1081 | 0.014191 | 1.848 |
| MALEATE | 0.38528 | -1.376 | 0.05722 | 1.2425 |
| Riboflavin | 0.48773 | -1.0359 | 0.054337 | 1.2649 |
| GLUCOSAMINE | 2.5663 | 1.3597 | 0.006135 | 2.2122 |
| Creatine | 2.9909 | 1.5806 | 0.035561 | 1.449 |
| Glucose | 6.1875 | 2.6294 | 0.013849 | 1.8586 |
| **HG, 5% O_2_ vs 21% O_2_** | | | | |
| 4-Pyridoxic acid | 0.05244 | -4.2532 | 0.001978 | 2.7038 |
| Uric acid | 0.23352 | -2.0984 | 0.048272 | 1.3163 |
| PE 36:3 | 0.2833 | -1.8196 | 0.084566 | 1.0728 |
| 4-Hydroxybenzaldehyde | 3.9644 | 1.9871 | 0.069715 | 1.1567 |
| Uracil | 4.2057 | 2.0723 | 0.072206 | 1.1414 |
| ETHYL SULFATE | 6.2389 | 2.6413 | 0.003194 | 2.4956 |
| Hypoxanthine | 16.807 | 4.071 | 0.006851 | 2.1642 |
| **LG, 5% O_2_ vs 21% O_2_** | | | | |
| 4-Pyridoxic acid | 0.091929 | -3.4433 | 0.021668 | 1.6642 |
| Cytidine 2',3'-cyclic monophosphoric acid | 0.38716 | -1.369 | 0.050428 | 1.2973 |
| Guanine | 5.7683 | 2.5282 | 0.009296 | 2.0317 |
| Hypoxanthine | 7.7285 | 2.9502 | 0.078904 | 1.1029 |
| ETHYL SULFATE | 7.977 | 2.9959 | 0.008906 | 2.0503 |

**Table S2:** Differentially regulated metabolites in subchondral bone co-cultured with cartilage and synovium under 21% and 5% O_2_ and high glucose (HG) and low glucose (LG) medium for 7 days.

| **HG vs LG, 21% O_2_** | | | | |
| --- | --- | --- | --- | --- |
| **Metabolites** | **FC** | **log2(FC)** | **raw.pval** | **-LOG10(p)** |
| N-Oleoyl-Phenylalanine | 0.21973 | -2.1862 | 0.007756 | 2.1103 |
| Docosahexaenoic acid | 0.28619 | -1.8049 | 0.097043 | 1.013 |
| Uracil | 0.30717 | -1.7029 | 0.060919 | 1.2152 |
| Eicosapentaenoic acid | 0.32653 | -1.6147 | 0.074021 | 1.1306 |
| Arachidonic acid | 0.36249 | -1.464 | 0.029427 | 1.5313 |
| Oleoyl-L-lysophosphatidic acid | 0.39282 | -1.3481 | 0.077693 | 1.1096 |
| Xanthine | 0.39468 | -1.3412 | 0.027004 | 1.5686 |
| Cytidine 2',3'-cyclic monophosphoric acid | 0.43005 | -1.2174 | 0.075814 | 1.1203 |
| Hypotaurine | 0.47437 | -1.0759 | 0.083897 | 1.0763 |
| O-Acetylcarnitine | 2.8516 | 1.5118 | 0.005345 | 2.272 |
| Glucuronic acid | 2.8886 | 1.5304 | 0.055454 | 1.2561 |
| Mannitol | 5.8621 | 2.5514 | 0.011107 | 1.9544 |
| Gluconic acid | 6.448 | 2.6889 | 0.023518 | 1.6286 |
| Glucose | 8.7496 | 3.1292 | 0.00373 | 2.4283 |
| **HG vs LG, 5% O2** | | | | |
| 6-Ketoprostaglandin F1 | 0.3622 | -1.4651 | 0.003925 | 2.4062 |
| Eicosapentaenoic acid | 0.37551 | -1.4131 | 0.017983 | 1.7451 |
| Uracil | 0.38288 | -1.385 | 0.061884 | 1.2084 |
| N-Oleoyl-Phenylalanine | 0.39652 | -1.3345 | 0.005011 | 2.3001 |
| OLEATE | 0.41952 | -1.2532 | 0.072024 | 1.1425 |
| Oleoyl-L-lysophosphatidic acid | 0.48093 | -1.0561 | 0.050449 | 1.2972 |
| Gluconic acid | 3.94 | 1.9782 | 0.024241 | 1.6154 |
| Glucose | 6.5626 | 2.7143 | 0.005715 | 2.243 |
| **HG, 5% O_2_ vs 21% O_2_** | | | | |
| 4-Pyridoxic acid | 0.02733 | -5.1934 | 0.001359 | 2.8669 |
| Adenine | 0.081189 | -3.6226 | 0.016001 | 1.7958 |
| Adenosine | 0.15999 | -2.644 | 0.031398 | 1.5031 |
| Inosine | 0.21202 | -2.2377 | 0.016448 | 1.7839 |
| Uridine | 0.23317 | -2.1005 | 0.058965 | 1.2294 |
| cAMP | 0.31547 | -1.6644 | 0.088069 | 1.0552 |
| Mannitol | 0.32654 | -1.6147 | 0.096037 | 1.0176 |
| Guanosine | 0.32928 | -1.6026 | 0.01599 | 1.7962 |
| O-acetylcarnitine | 0.42825 | -1.2235 | 0.023368 | 1.6314 |
| N-Oleoyl-Phenylalanine | 2.2618 | 1.1775 | 0.00533 | 2.2733 |
| Ethyl sulfate | 3.9566 | 1.9842 | 0.026346 | 1.5793 |
| **LG, 5% O_2_ vs 21% O_2_** | | | | |
| 4-Pyridoxic acid | 0.081231 | -3.6218 | 0.008296 | 2.0811 |
| Uracil | 0.2581 | -1.954 | 0.022509 | 1.6476 |
| Allantoin | 0.37127 | -1.4294 | 0.073715 | 1.1324 |
| Guanosine | 0.38531 | -1.3759 | 0.005856 | 2.2324 |
| L-Alanine | 0.41558 | -1.2668 | 0.057742 | 1.2385 |
| Methionine | 0.41793 | -1.2587 | 0.046715 | 1.3305 |
| Beta-alanine | 0.4301 | -1.2173 | 0.08368 | 1.0774 |
| Isoleucine | 0.4609 | -1.1175 | 0.093091 | 1.0311 |
| Leucine | 0.47486 | -1.0744 | 0.061062 | 1.2142 |
| Pseudouridine | 0.47951 | -1.0604 | 0.002421 | 2.6159 |
| Hypotaurine | 0.49365 | -1.0185 | 0.094056 | 1.0266 |
| Tryptophan | 0.49909 | -1.0026 | 0.030129 | 1.521 |
| Ethyl sulfate | 7.9572 | 2.9923 | 0.001433 | 2.8439 |

**Table S3:** Differentially regulated metabolites in synovium co-cultured with cartilage and bone under 21% and 5% O_2_ and high glucose (HG) and low glucose (LG) medium for 7 days.

| **HG vs LG, 21% O_2_** | | | | |
| --- | --- | --- | --- | --- |
| **Metabolites** | **FC** | **log2(FC)** | **raw.pval** | **-LOG10(p)** |
| 4-Hydroxybenzaldehyde | 0.064638 | -3.9515 | 0.04573 | 1.3398 |
| Uracil | 0.20494 | -2.2867 | 0.004539 | 2.343 |
| Adenine | 0.38519 | -1.3764 | 0.092891 | 1.032 |
| Indoxyl sulfate | 0.39087 | -1.3552 | 0.066435 | 1.1776 |
| PE 36:3 | 0.39326 | -1.3464 | 0.034306 | 1.4646 |
| Cystathionine | 0.46416 | -1.1073 | 0.025409 | 1.595 |
| Proline | 0.47847 | -1.0635 | 0.096215 | 1.0168 |
| Oxoglutaric acid | 6.4519 | 2.6897 | 0.000178 | 3.7492 |
| Gluconic acid | 16.248 | 4.0222 | 0.007504 | 2.1247 |
| Mannitol | 21.082 | 4.3979 | 0.010567 | 1.976 |
| Glucose | 27.886 | 4.8015 | 0.000565 | 3.2479 |
| **HG vs LG, 5% O_2_** | | | | |
| Methyl beta-D-galactoside | 0.27902 | -1.8416 | 0.063715 | 1.1958 |
| N-Acetyl-L-alanine | 2.2305 | 1.1574 | 0.015312 | 1.815 |
| Methionine sulfoxide | 2.5382 | 1.3438 | 0.087481 | 1.0581 |
| D-Alanine | 3.1097 | 1.6368 | 0.06285 | 1.2017 |
| Mannitol | 14.507 | 3.8587 | 0.032246 | 1.4915 |
| Gluconic acid | 20.188 | 4.3354 | 0.006813 | 2.1667 |
| Glucose | 20.233 | 4.3386 | 0.00021 | 3.6774 |
| **HG, 5% O_2_ vs 21% O_2_** | | | | |
| 4-Pyridoxic acid | 0.13107 | -2.9316 | 0.051091 | 1.2917 |
| Adenosine | 0.27024 | -1.8877 | 0.066161 | 1.1794 |
| Oxoglutaric acid | 0.29825 | -1.7454 | 8.14E-05 | 4.0893 |
| 5,5-dimethyl-2-{[(2-phenylacetyl)amino]methyl}-1,3-thiazolane-4-carboxylic acid | 2.2363 | 1.1611 | 0.001596 | 2.797 |
| Xanthine | 2.3017 | 1.2027 | 0.08963 | 1.0475 |
| 4-Oxoproline | 2.3391 | 1.2259 | 0.005165 | 2.2869 |
| Quinolinecarboxylic acid | 2.8288 | 1.5002 | 0.034318 | 1.4645 |
| D-Alanine | 3.8629 | 1.9497 | 0.028525 | 1.5448 |
| Glycerol 3-phosphate | 4.5189 | 2.176 | 0.06977 | 1.1563 |
| Ethyl sulfate | 10.106 | 3.3371 | 0.008218 | 2.0853 |
| **LG, 5% O_2_ vs 21% O_2_** | | | | |
| 4-Pyridoxic acid | 0.063457 | -3.9781 | 0.013486 | 1.8701 |
| Adenosine | 0.23005 | -2.12 | 0.030318 | 1.5183 |
| Guanosine | 0.23009 | -2.1198 | 0.063591 | 1.1966 |
| Adenine | 0.24346 | -2.0382 | 0.028188 | 1.5499 |
| 6-Ketoprostaglandin F1 | 0.24847 | -2.0089 | 0.085871 | 1.0662 |
| Methyl beta-D-galactoside | 0.31279 | -1.6767 | 0.071452 | 1.146 |
| Uracil | 0.3457 | -1.5324 | 0.060529 | 1.218 |
| Glycerol | 5.8899 | 2.5582 | 0.085104 | 1.0701 |
| Ethyl sulfate | 9.9798 | 3.319 | 0.000763 | 3.1173 |

**Table S4:** Baseline expression of glucose transporter genes in cartilage, subchondral bone, and synovium at day 0. Expression values are presented as mean ± SD of log₁₀(2^-ΔCt) measured in tissues prior to culture at day 0. *GLUT1*, *GLUT3*, *GLUT4*, and *GLUT5* expression levels were calculated relative to the reference gene (RPLP0).

| **Transporter** | **Cartilage** | **Bone** | **Synovium** |
| --- | --- | --- | --- |
| **GLUT1** | -0.900 ± 0.134 | -0.864 ± 0.145 | -0.912 ± 0.350 |
| **GLUT3** | -1.622 ± 0.306 | -0.250 ± 0.382 | -0.643 ± 0.381 |
| **GLUT4** | -4.186 ± 0.300 | -3.529 ± 0.217 | -3.237 ± 0.151 |
| **GLUT5** | -3.319 ± 0.461 | -3.957 ± 0.268 | -5.003 ± 0.557 |

**Figure S1:** Principal component analysis (PCA) of metabolomics data of (A) cartilage, (B) bone and (C) synovium cultured for 7 days in LG or HG medium under 21% or 5% O2 concentration.

**Figure S2:** Pathway analysis of the differentially expressed metabolites in (A) cartilage, (B) subchondral bone, and (C) synovium. Pathway analyses are performed separately on the list of metabolites that were differentially expressed in tissues cultured in HG vs LG under either 5% or 21% O2. Similarly, the analysis is performed for the differentially expressed metabolites in tissues cultured under 5% vs 21% either in HG or LG condition.

**Figure S3**: Normalized log2-transformed glucose levels in fresh and 7-day cultured tissues in (A) cartilage, (B) subchondral bone, and (C) synovium. Each dot represents an individual biological replicate. N=3. Control is the fresh tissue. Statistical analysis was performed using one-way ANOVA, with significance levels indicated as *p < 0.05, ****p < 0.0001.
